# Deciphering the roles of lncRNAs in breast development and disease

**DOI:** 10.18632/oncotarget.24591

**Published:** 2018-02-28

**Authors:** John Lalith Charles Richard, Pieter Johan Adam Eichhorn

**Affiliations:** ^1^ Cancer Science Institute of Singapore, National University of Singapore, 117599, Singapore; ^2^ Department of Pharmacology, Yong Loo Lin School of Medicine, National University of Singapore, 117597, Singapore; ^3^ School of Pharmacy, Curtin University, Perth, 6845, Australia; ^4^ Current Address: Genome Institute of Singapore, Agency for Science Technology and Research, 138672, Singapore

**Keywords:** long non-coding RNA, breast cancer, mammary gland development, XIST, HOTAIR

## Abstract

Breast cancer is the second leading cause of cancer related deaths in women. It is therefore important to understand the mechanisms underlying breast cancer development as well as raises the need for enhanced, non-invasive strategies for novel prognostic and diagnostic methods. The emergence of long non-coding RNAs (lncRNAs) as potential key players in neoplastic disease has received considerable attention over the past few years. This relatively new class of molecular regulators has been shown from ongoing research to act as critical players for key biological processes. Deregulated expression levels of lncRNAs have been observed in a number of cancers including breast cancer. Furthermore, lncRNAs have been linked to breast cancer initiation, progression, metastases and to limit sensitivity to certain targeted therapeutics. In this review we provide an update on the lncRNAs associated with breast cancer and mammary gland development and illustrate the versatility of such lncRNAs in gene control, differentiation and development both in normal physiological conditions and in diseased states. We also highlight the therapeutic and diagnostic potential of lncRNAs in cancer.

## INTRODUCTION

Breast cancer poses a major public health concern with breast cancer being not only the most common cancer in women but, also the second leading cause of cancer related deaths in women worldwide [1]. Even with the incredible recent surge in diagnostics and drug discovery, resistance to cancer treatments remains an altogether substantial issue [2]. Innumerable mutations and copy number changes in tumor suppressors and oncogenes have been implicated in breast cancer development leading to a cascade of events enhancing mammary cell proliferation, abnormal differentiation and enhanced migration and invasion [3, 4]. However, in a large subset of cancers the causal agents of breast cancer remain obscure. Interestingly, even though the mammary gland itself is unimportant for the maintenance of life, the onset of breast cancer can be fatal if the disease manifests and metastasis ensues. During mammary gland development from embryogenesis through puberty, pregnancy, lactation and involution, the mammary gland undergoes a series of morphogenetic cues arising from a complex crosstalk of molecular factors effecting dramatic anatomical and physiological changes in the gland [5–7]. Interestingly, the genetic factors required for the organization of this complex framework have also been demonstrated to be mutated in breast cancer.

The advent of sequencing technologies has enabled intense whole genome transcriptome analysis possible and our understanding of the components that make up our complex genome has been revamped [8–10]. Not too long ago the majority of the transcriptome realm was still considered “junk” because large parts of the DNA did not appear to transcribe functional proteins. However, recently these vast stretches of the genome have been annotated and discovered to contain thousands of non-coding RNA (ncRNA). Unexpectedly, ncRNA and its various species are more abundant than protein coding genes. Today, thousands of these ncRNA have been reported and catalogued, thanks to huge genomics initiatives like ENCODE (Encyclopedia of DNA Elements), FANTOM (Functional Annotation of Mouse Genome), GTEx (Genotype-Tissue Expression) and GENCODE, the field of ncRNA research has exploded [11–14]. Furthermore, exploration into the roles of ncRNA has vastly enhanced our understanding of human diseases including the pathophysiology of breast cancer [15, 16].

Non-coding RNAs are divided into two broad classes based on their functions, as housekeeping and regulatory. While, housekeeping ncRNAs are constitutively expressed and are particularly involved in the operation of vital cellular functions, regulatory ncRNAs are expressed in a developmental and tissue specific manner or may be altered in certain disease conditions. Some of the ncRNAs that fall under the housekeeping ncRNAs are ribosomal RNA (rRNA), transfer RNA (tRNA), small nuclear RNA (snRNA) and small nucleolar RNA (snoRNA). Additionally, regulatory ncRNA are broadly classified into two classes as short non-coding transcripts or long non-coding. Examples of short non-coding RNAs include microRNA (miRNA), small interfering RNA (siRNA), piwi interacting RNA (piRNA), among others [17–21]. Like short ncRNA, lncRNA has been demonstrated to play important roles in numerous diseases including cancer [22–25]. However, to date only but a small fraction of the lncRNAs have been functionally characterized, leaving us to ponder over the biological functions of those remaining and the crucial roles they might play. LncRNAs have been implicated in many biological processes like dosage compensation, imprinting, cell cycle regulation, retro-transposon silencing, pluripotency and telomere lengthening just to name a few. They further employ diverse molecular mechanisms to carry out multiple functions [26–29]. While some lncRNAs are involved in the regulation of genes through their interaction at the transcription site, others behave as molecular decoys, trapping transcription factors, diverting the association with DNA transcription factor binding sites. Numerous studies have reported altered lncRNA function in cancer with a number of these lncRNAs displaying unique tissue specific expression patterns. Mutations in lncRNAs like HOTAIR, MALAT1, ANRIL, h19 and PCA3 have been reported in a number of tumor types [23, 30–34]. Interestingly, lncRNAs can display altered expression patterns in breast cancer compared to normal tissues [35–39]. In spite of the growing research findings, aimed at understanding the molecular and cellular mechanisms involved in these pathologic responses, the functional aspects of the majority of lncRNA still remains uncharacterized. In this review, we provide an update of the lncRNAs that are associated with breast development and disease, highlighting the function of known lncRNAs in these areas.

### Long non-coding RNA

Using the length of non-coding RNA as a cut-off for its classification, conveniently sieves non-coding RNA into various categories such as miRNAs, siRNAs, snoRNAs, lncRNAs among other species [9, 40, 41]. LncRNAs as their name suggests, represents a class of non–protein coding transcripts which are defined as transcripts of greater than 200 nucleotides [42]. In some cases lncRNAs can also be referred to as macro lncRNA if they encompass lengths greater than 90kb such as the macro lncRNAs Air and KCNQ1OT1 [43, 44]. Table 1 highlights the specific types of lncRNAs annotated thus far.

**Table 1:** Types of long non-coding RNA

Long non-coding RNA	Representative symbol
Long intergenic non-coding RNA	LincRNA
Long Intronic non-coding RNA	-
Natural Antisense Transcript	NAT
Promoter associates long RNA	PALR
Promoter upstream Transcript	PROMPT
Transcribed Ultraconserved Region	T-UCR
Enhancer like non-coding RNA	eRNA
Circular RNA	circRNA

Many lncRNA have H3K27me3 methylation marks at their promoters as well as RNA PolII binding sites as an indicator of active transcription [45, 46] Many lncRNAs undergo RNA polymerase II mediated transcription followed by 5` capping, polyadenylation and even splicing [46, 47]. However, it is important to note that not all lncRNA are polyadenylated. A fraction of lncRNAs also have a H3K4me1 monomethylation mark as a sign of enhancer produced RNAs [48]. Additionally, lncRNAs can be readily identified by cross referencing high throughput RNA sequencing data with the promoter region of lncRNAs as they are enriched with histone marks such as H3K4me3, H3K9ac and H3K27ac [45, 46]. Interestingly, these histone marks are found around transcription start sites and have distribution patterns and densities correlating with protein coding and non-coding genes. Interestingly, lncRNAs share histone marks of active transcription similar to protein coding genes, however, they differ in repressive marks like DNA methylation and H3K9me3 histone modifications [49]. The sequences of lncRNAs, their transcriptional regulation, and the additional 3D structure of these transcripts accounts for their diverse functions [50].

LncRNAs constitute the largest proportion of the non-coding transcriptome. However, recently it is has been shown that certain lncRNAs can also form complexes with ribosomes as well as giving rise to small peptides [51–56]. Thus, distinguishing some lncRNAs from other non-coding RNA. The ability of some lncRNAs to code for small peptides indicates the potential for some lncRNA to act as bifunctional transcripts giving rise to either a lncRNA or a protein template [56–58].

**Figure 1:**
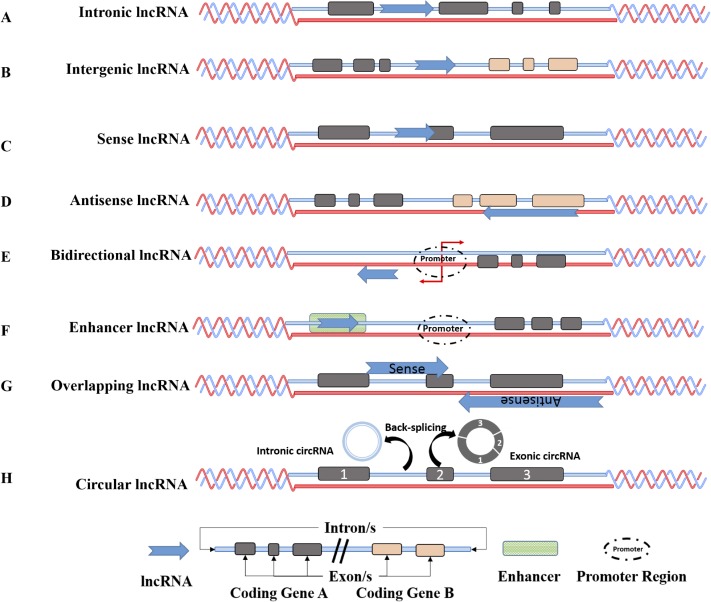
Genomic organization of long non-coding RNA Intronic lncRNAs are located in an intron of a coding region (**A**), Intergenic lncRNAs (lincRNAs are transcripts located between two protein coding genes (**B**), Sense lncRNAs are transcribed from the sense strand of the protein coding genes and might overlap one or several introns and exons (**C**), Anti sense (AS) lncRNAs are transcribed from the antisense strand of protein coding genes and could similarly overlap with one or several introns and exons of the sense strand (**D**) Bidirectional lncRNAs are located within 1 kb of promoters in the opposite direction of protein coding transcripts (**E**), and Enhancer lncRNAs are located in the enhancer regions (**F**). Sense-overlapping lncRNAs are lncRNAs that are present within the boundaries of the protein-coding gene. Some sense lncRNAs are transcript variants of protein-coding mRNAs and may not possess a functional open reading frame for translation into proteins. Antisense overlapping lncRNAs are transcribed from the antisense strand and overlap in part with well defined spliced sense or intronless sense RNAs and undergoes fewer splicing event and has lower abundance than sense transcripts (**G**). Circular RNAs are generated usually by backsplicing wherein a downstream splice donor is fused with an upstream splice acceptor. Such circRNAs can consist of one or more exons and can even contain unspliced intronic sequences (**H**).

LncRNAs can also be classified based on their genomic organization or their relation to protein coding genes as: 1) intronic lncRNAs (produced from an intron of a gene), 2) intergenic lncRNAs (existing between two protein coding genes), 3) sense lncRNAs (overlapping one or more exons of a coding gene and in the same direction and strand), 4) antisense lncRNAs transcripts (arising from the opposite strands having a partial or complete sequence complementarity, 5) bidirectional lncRNAs (transcribed in opposite directions but sharing the same promoter regions with protein coding genes), and 6) enhancer lncRNAs (eRNA- produced from enhancer regions of proteins) and circular RNA [28] (Figure 1).

Another fairly recent addition to the family of lncRNAs are the circular RNAs (circRNAs). CircRNAs are abundant ncRNAs that span a range of hundreds to thousands of base pairs [59]. Additionally, they are conserved, transcribed by RNA polymerase II, occur endogenously, and were primarily thought to arise as splicing byproducts. However, high throughput sequencing and advancements in computational techniques show that these covalently closed continuous loop circular RNAs can be of many types based on their origin and sequence composition, as exonic, circular intronic or retained-intron circRNA formed either by exon back splicing or exon skipping. CircRNAs are also highly tissue and cell type specific and been observed to be developmentally regulated [60, 61]. Though the biogenesis of circRNAs and its functional properties are still in the early stages of investigation, evidence points towards their biogenesis from circularization of single or multiple exons or a combination of an intron and exon or simply the intronic sequences alone [62–68].

**Figure 2:**
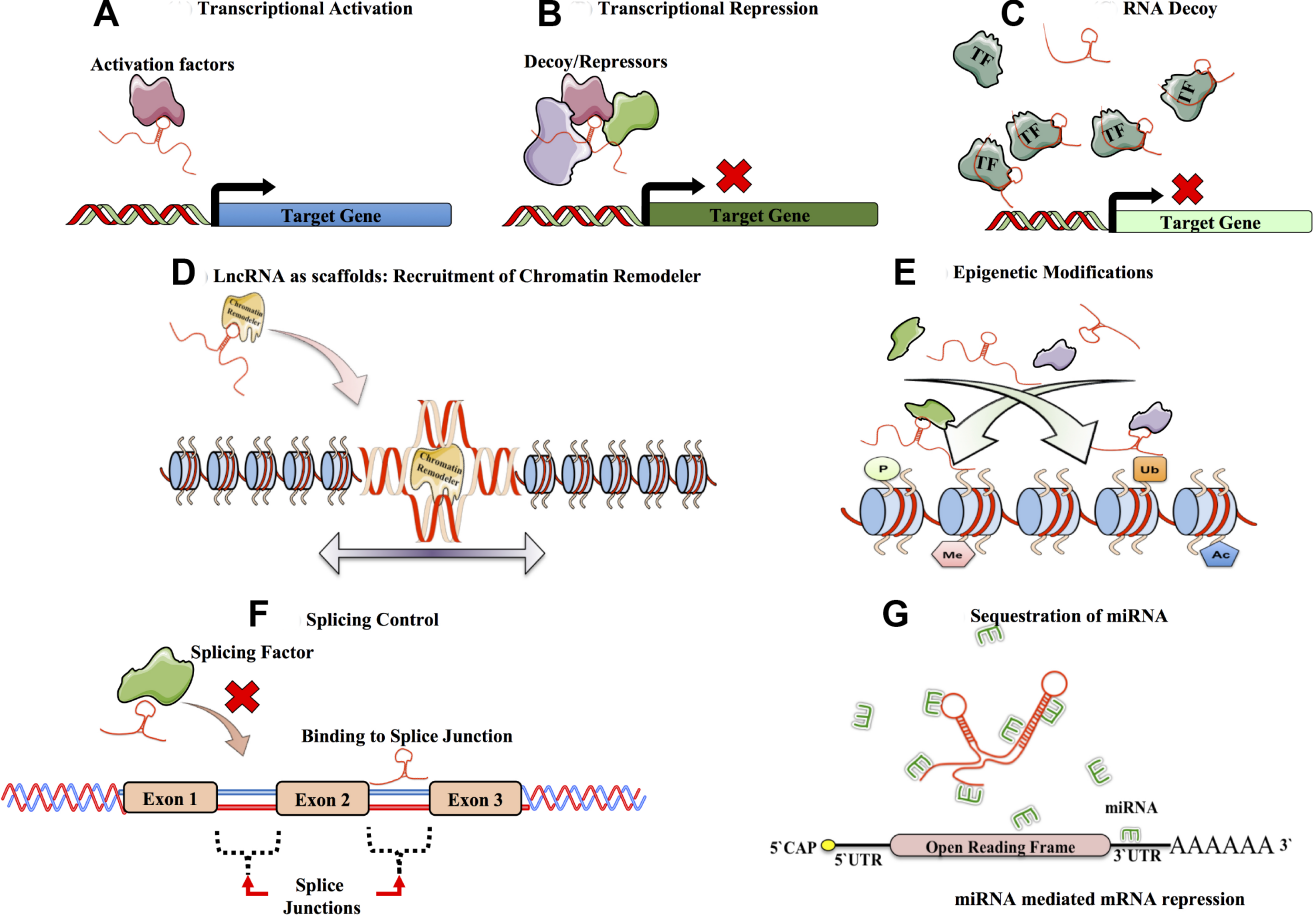
Functions of long non-coding RNA Long non-coding RNA, have diverse mechanisms of functions mainly owing to their sequence, 3D structures and diverse interacting partners. Some of the functions are as follows. LncRNAs interact with transcriptional activators and are involved in target gene activation (**A**). Likewise lncRNAs have also been shown to be involved in transcriptional repression by preventing transcriptional activators from accessing the promoter (ex. XIST or SRG1) (**B**). Additionally, lncRNAs behave as decoys sequestering transcription factors or other activation complexes interfering and directly affecting or regulating the signaling pathways by diluting them from their target regions (ex. GAS5 or H19) (**C**). Additionally they can also recruit transcriptional repressors and ultimately lead to transcriptional repression of target genes. lncRNAs also serve as scaffolds, providing a platform for recruiting chromatin remodelers and other ribonucleoproteins (RNPs) directing changes in the chromatin structure (ex. HOTAIR or Six3OS). (**D**). LncRNAs are also involved in bringing about epigenetic changes in the chromatin. The epigenetic changes (activation or repression marks) brought, depends on the epigenetic marks such as phosphorylation, acetylation, ubiquitination or methylation (ex. XIST and HOTAIR) (**E**). LncRNAs are involved in regulating RNA splicing by interacting with the splicing factors or by binding the splicing junctions of pre-mRNA (ex. asFGFR2) (**F**). LncRNAs also serve as molecular sponges by harboring binding sites for miRNAs and titrating or diluting them away from their miRNA targets (ex. linc-ROR and H19) (**G**).

The wide interactome of lncRNA elucidates to their diverse role in transcription, chromosome remodeling and intra-cellular trafficking. Furthermore, lncRNAs have been demonstrated to be involved in more functions such as transcriptional regulation, mRNA processing, mRNA maturation and epigenetic regulation through lncRNA-mediated recruitment of chromatin remodelers, histone modifying complexes and DNA methyltransferases [26, 42, 69–71]. Figure 2 highlights the various functions of lncRNA in cellular signaling. Initially, lncRNAs were thought to predominantly have a nuclear function and only until recently, where reports noted their presence in the cytoplasm, has a potential role for lncRNAs in post transcriptional gene regulation, protein and transcript trafficking and shuttling been suggested [72]. Though studies pertaining to lncRNAs is still in its infancy, with only a handful of lncRNAs having been studied in detail to date, growing evidence points to their diverse role in development both in terms of tissue specificity and developmental stage [73, 74] (Table 2).

**Table 2:** Long non-coding RNAs in breast cancer and their genomic organization

lncRNA in breast cancer	Genomic organization
ARA, BC200, CCAT2, SPRY4-IT1	Intronic lncRNA
H19, MALAT1, MEG3, LincROR, HOTAIR, XIST, UCA1, LINC00324, LSINCT5	Intergenic lncRNA
XIST	Sense lncRNA
HOTAIR, ANRIL, ZFAS1	Antisense lncRNA
H19, SOX2OT	Overlapping lncRNA
CUPID1 and CUPID2	Bidirectional lncRNA
P53-eRNA	Enhancer lncRNA
Circ-ABCB10	Circular lncRNA

A number of recent reports have highlighted the differential expression patterns of lncRNAs between cancer cells versus normal cells [75–78]. Furthermore, lncRNAs can alter the activity of different hallmarks of cancer by either sustaining proliferative signaling (e.g. SRA), evading growth suppressors (e.g. ANRIL), inducing angiogenesis (e.g. MVIH), inducing invasion and metastasis (e.g. HOTAIR and MALAT1), inhibiting apoptosis (MEG3), or by enabling replicative immortality (TERC and TERRA) [23, 79–82]. For instance, the p53 pathway is regulated by the lncRNAs MEG3, MALAT1 and WRAP53 (a natural antisense transcript of p53), in a DNA damage dependent and independent manner [83–88]. Additionally, some lncRNAs are induced by p53 like lincRNAs p21, PANDA, ROR, H19 and Loc285194 all stimulating downstream p53 mediated cell cycle arrest or apoptosis [89–93]. The roles of lncRNAs towards p53 regulation showcases the complexity of the lncRNA interaction network.

Interestingly, genome wide association studies (GWAS), performed on various cancers can help us understand the genetic association of lncRNAs and cancers. It is worthy to note that single nucleotide polymorphisms (SNPs) can be involved in neoplastic progression. Importantly, a huge fraction of the SNPs lies within the non-coding intronic and intergenic regions of the genome harboring functional lncRNAs. One such interesting GWAS hotspot is the genomic locus giving rise to the lncRNA ANRIL. ANRIL is a 126 kb lncRNA located adjacent to INK4A/ARF locus and is linked to a number of diseases such as cancer, type-2 diabetes and coronary artery disease [94]. ANRIL interacts with the polycomb group of proteins establishing a repressed state at the INK4A/ARF locus [94–96]. Intriguingly, SNPs associated with ANRIL are phenotypically clustered, with SNPs attributed to causing vascular conditions appearing at the 3` end of the ANRIL transcript while cancer susceptibility SNPs are found at the 5` end of the transcript. The specific localization of SNPs along the ANRIL transcript and the altered phenotypes which are presented indicate the complexity of lncRNA function in the genome. While these SNPs may influence lncRNA stability they may also limit transcription factor binding to the INK4A/ARF locus or function in *cis* to regulate gene expression at other loci. It is this complexity that both continues to frustrate and intrigue. Nevertheless, a better understanding of lncRNA function will undoubtedly lead to better diagnostic and potential therapies [94].

### Mammary gland development and breast cancer

**Figure 3:**
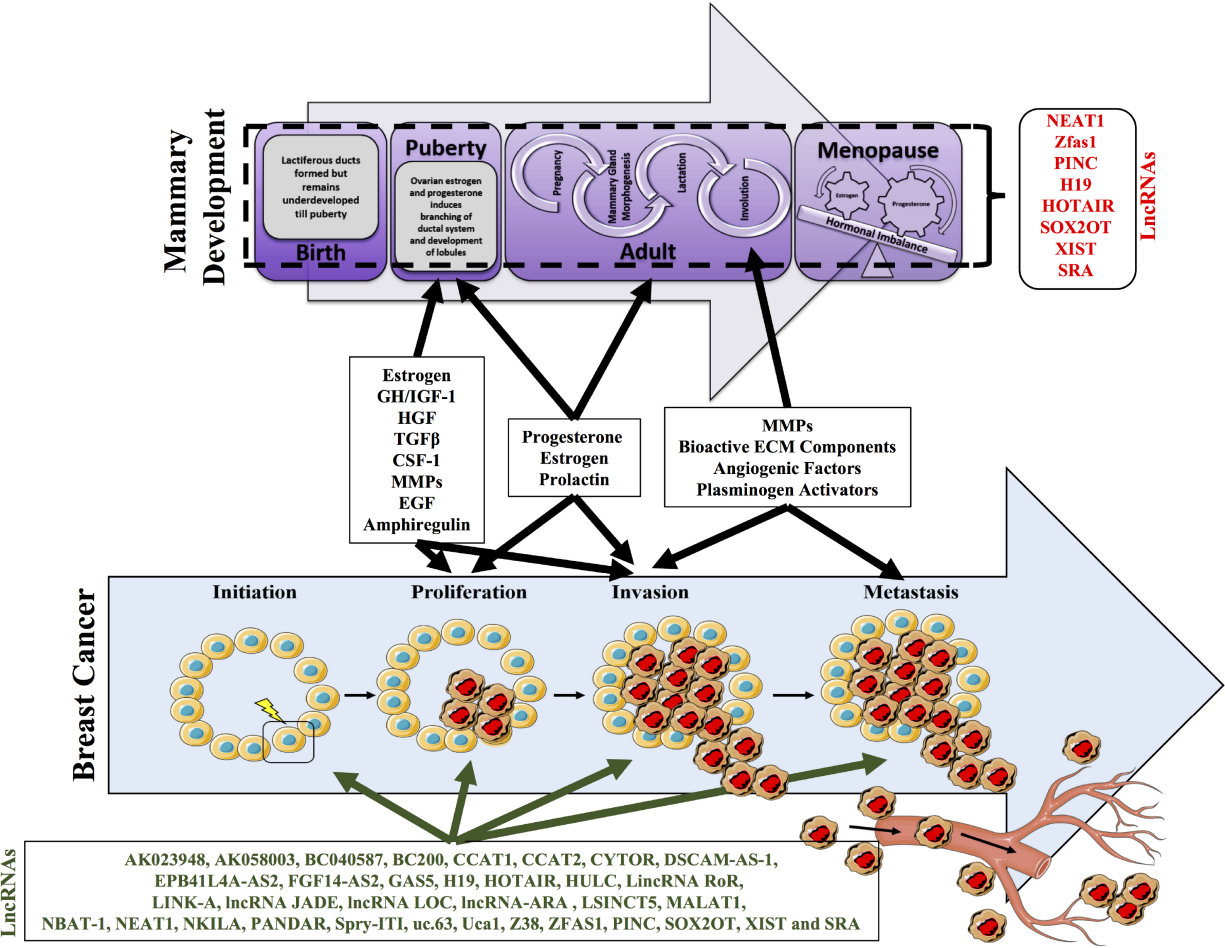
Mammary development with respect to the developmental stages Postnatal mammary gland development is composed of several stages and undergoes tremendous remodeling and morphogenesis. Highlighted are the key changes that occur at birth, puberty, adult and menopause. At birth lactiferous ducts are formed but remain underdeveloped until puberty. At puberty ovarian estrogen and progesterone induces the branching of ductal systems and development of lobules. The adult stage marks an important stage of development wherein the onset of pregnancy triggers mammary gland morphogenesis. Additionally, milk ducts are formed that emanate into the nipple. Subsequently post pregnancy, involution occurs wherein all the lactiferous ducts are disintegrated. The onset of menopause can impact on the tightly regulated hormonal levels and can tip off an imbalance in the hormonal levels. A number of lncRNAs have suggested to be involved in these processes including, NEAT1, ZFAS1, PINC, H19, XIST, SRA, HOTAIR, AND SOX2OT). Importantly alterations in the factors required for mammary gland development and involution, may further modify the ability of cells to undergo neoplastic transformation required for breast cancer progression and metastasis.

During mammary gland development and pregnancy the mammary glands undergo dramatic changes in glandular morphology and cellular functions. During embryogenesis, these transformations are directed by signals from the surrounding mesenchyme, but during puberty and into adulthood, circulating hormones released from the pituitary and ovary provide additional instructive input [5]. The human breast consists of a mélange of parenchymal and stromal elements. While the former gives rise to the branching ducts, which eventually leads to the development of secretory acini, the later consists mainly of adipose tissues involved in providing a suitable environment for parenchymal development [5, 97–99]. In puberty, hormonal stimulation initiates further differentiation of acini branching morphogenesis, which is halted in early childhood to develop into a mature mammary gland [100, 101]. Intriguingly, the mammary gland undergoes immense rounds of development and differentiation, which are linked to the distinct stages of sexual development from puberty to adulthood and reproduction. A mixture of different cell types, comprising epithelial cells, adipocytes, vascular endothelial cells and stromal cells converge to form the secretory organ, the mammary gland. During mammary gland development these different cell types engage in an extensive crosstalk mediated by hormones, growth factors, and cytokines to ensure correct organization of the breast (Figure 3). Adipocytes are a major constituent of the stromal fat pad in mature non-lactating mammary glands. Furthermore, adipocytes present in the mammary gland also express aromatase enzymes that help in the conversion of the androgens to estrogen exhibiting a paracrine effect on estrogen receptor-a positive epithelial cells. Additionally, adipocytes regulate angiogenesis in the mammary gland by the secretion of VEGF- vascular endothelial growth factors. Apart from adipocytes found in the mammary gland another principal component of the mammary gland is fibroblasts. Fibroblasts secrete growth factors, which in turn regulate the epithelial cell survival, proliferation and differentiation during morphogenesis. Fibroblasts can maintain the extra cellular matrix (ECM) through collagens, proteoglycans and fibronectins whilst simultaneously leading to the degradation and remodeling of the ECM by expressing enzymes such as matrix metalloproteinases.

This highlights the remarkable plasticity of cells within the mammary gland. However, this is never truer than during pregnancy where the breast undergoes a cyclic transformation wherein the non-functioning gland matures into an organ producing milk. It returns back to a normal resting non-functional mammary gland once the production of milk is ceased (Figure 3). This is also referred to as the pregnancy lactation cycle (PLC) and has been shown to alter the molecular histology of the breast with tremendous cellular remodeling, morphological changes including cell death during involution.

It has been observed in mouse models that involution post lactation is a complex process involving more than 50 differentially expressed genes regulating a myriad of distinct molecular pathways and developmental processes [102]. During postlactational involution, the dissolving of tissue architecture along with the additional activation of factors within the tumour microenvironment could lead to the development of premalignant cells in mammary gland [103]. When the extracellular matrix isolated from mammary glands was compared between nulliparous or the postlactational mice, tumorigenic ECM fragments that promote outgrowth of breast cancer cells in culture were found in the mammary glands undergoing remodeling [104–107]. This additionally promoted breast cancer metastasis in animal models [104–107]. While these model systems have demonstrated an increased likelihood for tumor developmental, data supporting this is very sparse [106, 107]. It is more than likely that any significant delay in the postlactational involution may increase cancer formation risk potentially through the deregulation of microenvironmental influences within the mammary gland [103, 108]. Intriguingly, cancer linked to lobular involution may be an age related protective process with early pregnancies demonstrating a statistically significant lower incidence of breast cancer versus pregnancies in older women [109, 110]. Nevertheless, nulliparous women have a 20%–40% higher risk of postmenopausal breast cancer than parous women who first gave birth before age 25 [111]. A potential simple explanation for the reduced risk for breast cancer is the removal of the epithelial cells during the involution process thus eliminating any progenitor cancer stem cells required for the formation of the tumors [110, 112].

Hormones are critical factors in mammary development. A spike in the levels of progesterone and estrogen during pregnancy is observed with the concentrations of both hormones decreasing rapidly post-partum [113, 114]. This splurge in hormone levels during pregnancy may increase the risk of breast cancer in the short term, in particular for certain hormone sensitive cancer subtypes. However, the mechanisms governing the protective long-term anti-cancer effect is likely to be more complex with both age of first pregnancy and efficient involution of the terminal duct lobular units being just two of the factors involved in neoplastic growth. In addition, elevated levels of testosterone and estrogens have been documented in postmenopausal women and shown to positively correlate with increased breast cancer risk. Increased levels of androgens during pregnancy has also been associated with shorter breast feeding durations [115]. Interestingly, high levels of circulating androgens can be used as a biomarker for increased cancer risk in premenopausal women [116–118]. Additionally, elevated levels of prolactin, a hormone involved in normal breast development and lactation, is associated with a slight increase in breast cancer risk in both pre and postmenopausal women and is important in the etiology of breast cancer [119, 120]. Listed above are just some of the factors regulating mammary gland development and likewise breast cancer tumorigenesis. Furthermore, it is important to note that breast development is regulated by a transcriptional cascade that enables the concerted action of a number of cellular developmental processes with intrinsic or acquired alterations to any of the genetic components involved in these processes potentially driving breast cancer progression.

Breast cancer, as previously mentioned is a very heterogeneous disease and a number of key factors and biological features are considered for prognostic, diagnostic and predictive purposes. Many of such practices include histological grade, hormone receptor status, and lymph node status. With respect to gene expression, breast cancer is classified into 5 subtypes namely luminal A, luminal B, basal-like (also known as triple negative breast cancer), ERBB2+, and normal breast-like subtype [121]. The majority of these revolve around estrogen receptor (ER) and or progesterone (PR) receptors status, with the ER and PR positive tumors usually falling under the luminal subtypes further bifurcating into the two major classes luminal A and luminal B based on their Ki67 expression levels [122]. Like ER and PR, the ERBB2+ subtype or HER2+ subtype as it is commonly known is characterized by overexpression of the HER2 receptor. Tumors lacking all three receptors (ER-, PR-, HER2-) are classified into a triple negative subtype. Interestingly, the normal like subtype is characterized by its gene expression pattern, which is similar to that of normal breast but could have neighboring cancer cells infiltrating into its cellular matrix [123, 124]. A thorough and detailed diagnosis of these subtypes helps in associating the patient to the treatment regimen they should receive. Interestingly, patients falling under the same combination of features mentioned above can still follow different clinical paths restricting breast cancer therapy and emphasizing the need of personalized treatment.

### Non-coding RNA in mammary development

Mammary development remains an important field of research owing to the different stages of differentiation and remodeling. It is therefore important to dissect the pathways that control mammary development and differentiation at the cellular level. Irrespective of the immense research carried out towards understanding mammary development recapitulating the architecture and physiological intricacies during pregnancy still remain vague. More than 100 genes, including hormone directed regulation of specific proteins and other signaling pathway related genes are said to be involved in controlling mammary physiology from remodeling to involution. Interestingly, research carried out on the mouse mammary gland has elucidated the precise roles of some of transcription factors involved in or promoting the differentiation of luminal progenitors into ductal or alveolar cells [125]. Oncogenic initiating events targeting particular progenitor cells during mammary gland development through the action of such transcriptional regulatory networks could give rise to different outcomes with respect to the cancer subtypes [126, 127].

As mentioned, many coding genes have been well documented with regard to their roles in regulating embryonic, post-natal and post pubertal mammary gland development [126, 128–130]. The role of miRNAs in mammary gland development has been extensively studied, with a comprehensive miRNA profiling of the postnatal mammary gland having been carried out in mouse covering all the major stages of development including juvenile, puberty, mature virgin, gestation, lactation and involution stages [131]. This study highlights the importance of ncRNA in mammary development. A recent study also showcased a panoramic view of the lncRNAs in the bovine mammary gland and suggested their association with biological functions and also their involvement and susceptibility to clinical mastitis and eventually their milk quality and milk production. Such extensive annotation of the mammary gland and associated lincRNAs helps understand the bovine mammary gland biology [132]. However, the role of lncRNAs in the development of the mammary gland remain largely unexplored. Unlike proteins and miRNAs, lncRNAs are much less annotated and characterized. Interestingly, a few lncRNAs have been documented in literature as having a potential role in mammary gland development and are listed below (Figure 3).

Nuclear Paraspeckle Assembly Transcript 1 (Neat1) is an important architectural component of the paraspeckle nuclear bodies. Neat-1 paraspeckes have previously been identified as key regulators of gene expression effecting transcription factors and hyperediting mRNAs, however, it still remains unknown as to whether they have a biological relevance or under what physiological conditions these parascpeckles are formed *in vivo.* Standeart and colleagues showed that these parascpeckles are assembled in the luminal epithelial cells during mammary gland development [133]. Interestingly, the ablation of Neat1 results in an abnormal mammary gland morphogenesis and additional defects in lactation as well. The authors observed that this lactation defect is caused by the decreased ability of Neat1 mutant cells to sustain high rates of proliferation during lobular-alveolar development. This pioneering study shows the striking role of a lncRNA in mammary gland development and lactation in mice and paved way for further investigation.

H19 is highly expressed in alveolar cells during pregnancy and involution. H19 is an imprinted gene and has been demonstrated to be developmentally regulated with transcript levels increasing during both puberty and pregnancy [134]. Additionally H19 is also expressed in the uterus and is particularly high during the estrus and metestrus phases [134]. This same group demonstrated regulation of H19 by estradiol and corticosterone, both *in vitro* and *in vivo,* and in MCF-7 cell lines. Increased H19 levels were also detected in ovariectomize and adrenalectomised mice [134].

SRA (Steroid receptor RNA activator) is activated by steroid hormones and has been suggested to play a role in post pubertal mammary gland development [135]. The generation of a transgenic SRA mouse model where expression of the SRA gene was under the mouse mammary tumor virus long terminal repeat (MMTV) promoter demonstrated SRA expression in the nuclei of luminal epithelial cells of the mammary gland [135, 136]. The authors observed that although early ductal outgrowth was comparable between wildtype and SRA-transgenic strains in the adult virgin mice enhanced SRA expression led to an abnormal branching morphology in the transgenic duct. Furthermore, during pregnancy, progression of lobulo-alveolar structures was potentiated compared to control mice. However, while a strong correlation does exist between different physiological roles of steroid receptors, in particular the progesterone receptor, and the RNA transcript SRA in the regulation of development, the role of SRA in mammary gland development remains inconclusive as their RNA levels are barely detectable in virgin mouse mammary glands [135].

Zfas1 has recently been described as a novel regulator of mouse mammary development. Zfas1 is a transcript that is differentially expressed antisense to the 5` end of the protein coding gene ZNFX1. Zfas1 is localized within the ducts and alveoli of the mammary gland and hosts three snoRNAs (SNORDs): Snord12, Snord12b, and Snord12c [137]. Furthermore, Zfas1 transcripts are highly stable with their knockdown leading to increased cellular proliferation and differentiation without affecting overall SNORD levels. The human ortholog ZFAS1 has similar features to Zfas1, wherein both are highly expressed in the mammary gland and downregulated in tumors highlighting the potential tumor suppressor gene properties along with their regulatory role in alveolar development and epithelial cell differentiation [137, 138]. Interestingly it has recently been shown ZFAS1 is associated with ribosomes and could play a role in regulating their assembly or biosynthetic activity. Expression levels of ZFAS1 and ribosomal protein coding mRNAs involved in ribosome assembly, production and maturity have a tight correlation, with the knockout of ZFAS1 reducing RPS6 phosphorylation significantly [139].

Pregnancy induced Non-coding RNA (PINC) is a developmentally regulated non-coding RNA that is expressed in the parous mammary gland at the regressed terminal ductal lobular unit like structures [140]. Thus, PINC and ZFAS1, are both expressed during post pubertal mammary gland development, particularly in the alveolar cells during pregnancy. Interestingly, both these lncRNAs have decreased expression during lactation and an increased expression during early involution. Signifying that PINC expression is likely to be temporally and spatially regulated in response to developmental stimuli. One proposed mechanism of the role of PINC in mammary gland development is through the regulation of chromatin modifying complexes. PINC interacts with the retinoblastoma associated protein 46 (RbAp46), a component of the polycomb repressive complex 2 (PRC2), repressing gene expression. Although both PINC and RbAp46 are both upregulated in alveolar cells of the mammary gland during pregnancy it is now known that PINC likely functions to inhibit terminal differentiation of alveolar cells during pregnancy to prevent abundant milk production and secretion until parturition [141].

Hox Transcript antisense intergenic RNA (HOTAIR) is localized within the HoxC gene cluster. During mammalian embryonic development Hox genes, which are highly gene dense regions, are tightly regulated by the lncRNA HOTAIR. HOTAIR has been documented to downregulate HoxD gene expression which has previously been shown to be required for mediating the differentiation of the mammary epithelium ductal system in response to pregnancy [142]. Interestingly, HOTAIR functions in trans by increasing PRC2 recruitment to the genomic regions of target genes, repressing gene transcription [143]. A complete overview of HOTAIR and its role in chromatin dynamics has been extensively reviewed by Bhan and colleagues [144]. It is important to note here that the knockout of HOTAIR does not have an evident phenotype in the mouse mammary gland even though the PRC2 complex has been predicted to maintain a population of alveolar differentiated cells in the involuted gland. Furthermore, the actions and effects of Hotair, though widely studied, remain controversial. Targeted deletion of a 4 kilobase region within the HoxC cluster results in a severe skeletal phenotype. In contrast mice harbouring a complete deletion of the HoxC locus display no abnormal skeletal abnormalities or any changes in HoxD expression [145, 146]. Indicating that the complexity of this locus and the specific function of HOTAIR in this regard remains undetermined. HOTAIR has been observed to be involved in the regulation of cancer cell proliferation and cancer invasion in breast cancer which could arise from the positive correlation between HOTAIR and Hoxc11 genes [146]. Interestingly, HOTAIR is also regulated by estrogen and acts through estrogen receptors in ER+ breast cancer patients eluding to a feedback loop between the lncRNA and hormonal levels in normal individuals [147].

SOX2OT harbors the Yamanaka transcription factor SOX2 and is essential for maintaining pluripotency in a variety of stem cells. It is highly functional during embryonic development and is involved in stem cell maintenance. SOX2 and SOX2OT are differentially expressed in estrogen receptor positive and negative breast cancer tissues. SOX2 has also been shown to be critical determinant in mammary development [148]. Interestingly, the ectopic expression of the overlapping transcript leads to a massive increase in SOX2 expression levels, suggesting it plays a key role in the maintenance of SOX2 expression in breast carcinoma [149]. However, like HOTAIR as of yet there is no direct link of SOX2OT involvement in mammary gland development, but due to their critical roles in embryonic development and links to breast cancer they have included here.

In addition to these 6 candidate genes, a study performed on core needle biopsies from 42 nulliparous and 71 parous postmenopausal women analyzed differentiation associated gene expression within these two groups [150]. Interestingly the nuclei of epithelial cells were large and euchromatic in nulliparous breast cells and small and hyperchromatic in parous postmenopausal breast cells. Transcriptomic analysis recorded around 267 upregulated probe sets comprising genes involved in chromatin condensation, transcription regulation, splicing machinery and expectantly a number of non-coding RNA elements such as XIST, NEAT1, MALAT-1, CXorf50B, NCRNA00173 and NCRNA00201 [150]. These lncRNA molecules are all known to recruit the polycomb group of proteins that lead to the condensation of chromatin. MALAT-1 is upregulated by oxytocin during lactation, and both oxytocin specific receptor OTR and MALAT-1 are both upregulated in the breast of postmenopausal women, including in conditions where the circulating oxytocin is absent [150]. Similarly, XIST is upregulated in X chromosome inactivation upon differentiation. XIST repression occurs during early embryogenesis in undifferentiated ES cells by the binding of NANOG, OCT4 and SOX2 to the XIST gene, a similar phenomenon is exhibited in malignancies [151, 152]. Up-regulation of DDX, SOX1, SOX6 and SOX17 in postmenopausal nulliparous breast may also play a role in the regulation of XIST transcription similar to the pluripotency factors [150]. Table 3 summarizes the role of lncRNAs involved in mammary gland development. However, it is important to mention here that the majority of data summarized above extrapolates the potential of lncRNAs in mammary development based on correlative expression data with only a few lncRNAs having been directly implicated in mammary gland development.

**Table 3:** Non-coding RNA in mammary development

Non-Coding RNA	Category	Function	Reference
XIST	lncRNA	Differentially expressed in transcriptomic analysis between nulliparous and parous post-menopausal women. Important in embryonic development Recruitment of polycomb group of proteins.	[150]
NEAT1	lncRNA	Differentially expressed in transcriptomic analysis between nulliparous and parous post-menopausal women. Recruitment of polycomb group of proteins.	[150]
MALAT1	lncRNA	Differentially expressed in transcriptomic analysis between nulliparous and parous post-menopausal women. Recruitment of polycomb group of proteins.	[150]
H19	lncRNA	Regulated by estradiol and corticosterone. Highly expressed in alveolar cells during pregnancy and involution. Function in mammary gland development still unclear.	[134]
SRA	lncRNA	Activated by steroid hormones. SRA overexpression leads to cellular proliferation and differentiation accompanied by apoptosis but doesn't progress to malignancy. Function in mammary gland development still unclear.	[135]
ZFAS1	lncRNA	Suppresses mammary epithelial cell proliferation and differentiation. Expressed in post pubertal mammary gland development particularly in alveolar cells during pregnancy.	[137, 248]
PINC	lncRNA	Expressed in parous mammary gland at the regressed terminal duct lobular unit like structures. Expressed in post pubertal mammary gland development particularly in alveolar cells during pregnancy.	[140]
NCRNA00201	lncRNA	Differentially expressed in transcriptomic analysis between nulliparous and parous post-menopausal women. Function in mammary gland development still unclear.	[150]
NCRNA00173	lncRNA	Differentially expressed in transcriptomic analysis between nulliparous and parous post-menopausal women. Function in mammary gland development still unclear.	[150]
HOTAIR	lncRNA	Is dispensable in mouse mammary development. Is induced by estrogen through GPER receptors. Function in mammary gland development still unclear.	[146, 147]
SOX2OT	lncRNA	Increases SOX2 expression and may play a role in maintaining pluripotency in mammary stem cells. Function in mammary gland development still unclear	[149]
CXorf50B	lncRNA	Differentially expressed in transcriptomic analysis between nulliparous and parous post-menopausal women. Function in mammary gland development still unclear.	[150]

### LncRNAs in breast cancer

Although the roles of lncRNA are too numerous to discuss individually Supplementary Table 1 covers some of the lncRNAs that have been implicated in breast cancer. Nevertheless, we will discuss in detail the roles of the some of the lncRNAs involved in breast cancer below. It is important to indicate that a number of inconsistencies between various studies were noted potentially arising from the bioinformatics pipelines used in the individual studies, the different use of cell lines, or the different use of sequencing technologies. These inconsistencies are likely to be also dependent upon the intrinsic genetic heterogeneity of individual tumors.

X inactive Specific Transcript (XIST) is one of the most well studied long non-coding RNAs. Initially discovered in the early 90's it has been implicated in breast cancer progression. This lncRNA arises from the X inactivation center (XIC) nested within the X chromosome and which plays an important role in X chromosome inactivation [153]. Interestingly, many have reported the XIST expression correlates with the absence of a detectable Barr body in somatic cells, a strong cytological marker of the inactive X chromosome (Xi) [154, 155]. Furthermore, in human cancers a deregulation of XIST expression along with the loss of the normal X chromosome has been observed [156, 157]. XIST has also been shown to interact with BRCA1, a gene frequently mutated in triple negative breast cancers (TNBC) further implicating its role in breast cancer [158–162]. However, another group was able to show that in spite of increased copy numbers of the X chromosome, none of them are inactivated, irrespective of the BRCA1 status [163]. While the loss the Xi and the replication of active X chromosome (Xa) was observed in sporadic cancers and basal like breast cancers they were rarely seen in non-basal like breast cancers [164]. The role of BRCA1 and the localization of XIST on the Xi was widely debated and this heterogeneity of X chromosome inactivation (XCI) status was later attributed to the increased order of X chromosome instability, in addition to the loss of Xi [159, 165–167]. As mentioned XIST is crucial for XCI an effect accomplished through the recruitment of the PRC2 complex and induction of histone condensation and transcriptional inhibition [168–172]. X chromosome inactivation over the past decades has propelled our understanding of how XIST is transcribed, localized, silenced and eventually maintained [168, 169].

A number of factors are required for these steps to be establish on the inactive X chromosome. Recent work has established that HNRNPU, also known as SAF-A, anchors the lncRNA to the X chromosome while, SHARP recruits HDAC3 permitting PRC2 components to introduce repressive histone marks [173–177]. The recent high throughput studies of XIST interactors and regulators of XCI use varying techniques have further identified many more potential XIST interactors [173, 174, 178–181]. Intriguingly, there are discrepancies in the number of proteins pulled down, owing mainly to the strategies used, the crosslinking techniques and the cell lines used. BRCA1, being a tumour suppressor was observed in many cases to be mutated in patients with breast and ovarian cancer and was proposed to support the concentration of XIST on the inactive X chromosome [160]. However, this remains widely disputed with studies that followed showing that BRCA1 mutated primary breast tumours could still have one or more XIST RNA coated chromosomes [159, 166]. While these studies do not completely eliminate the role of BRCA1 in X inactivation, it still may be that BRCA1 could impact transcriptional regulation on a genome wide level by acting as a transcriptional regulator [182]. Additionally, while BRCA1 does not possess a consensus DNA binding sequence, it does contain many transcription factor binding sites suggesting the presence of BRCA1 at specific promoters as part of an inactive. BRCA1 would then revert to an active complex through the activation of certain stimuli like those stemming from DNA damage responses [182]. Another important chromatin modulator Aurora B Kinase has also been linked to cancer susceptibility owing to its role in chromosome segregation and cell division [183]. Aurora Kinase B regulates XIST RNA binding to the heterochromatin through phosphorylation of H3, regulating its association with the inactive X chromosome by means of anchor points within XIST [184]. A fairly recent study by Heard and colleagues showed that breast cancer cell lines and breast tumours exhibited abnormal 3D nuclear organizations and perturbations on a global scale. This was marked by an increase in euchromatic marks and an abnormal distribution of H3K27me3 repression marks along with increased DNA methylation at the promoters. Reactivation of many genes leading to epigenetic instability was observed in primary breast tumours and the disappearance of the epigenetic marks that build up on the inactive X chromosome leading to the disappearance of the Barr body in breast cancer cells [185]. The aggressiveness of the cancer though marked by the disappearance of the Barr body could correspond to the reactivation of the lncRNA or simply to its genetic loss. The status of XIST and the exact consequences therein on the Xi still remains unclear and is poorly understood with respect to cancer and requires further investigation.

**Figure 4:**
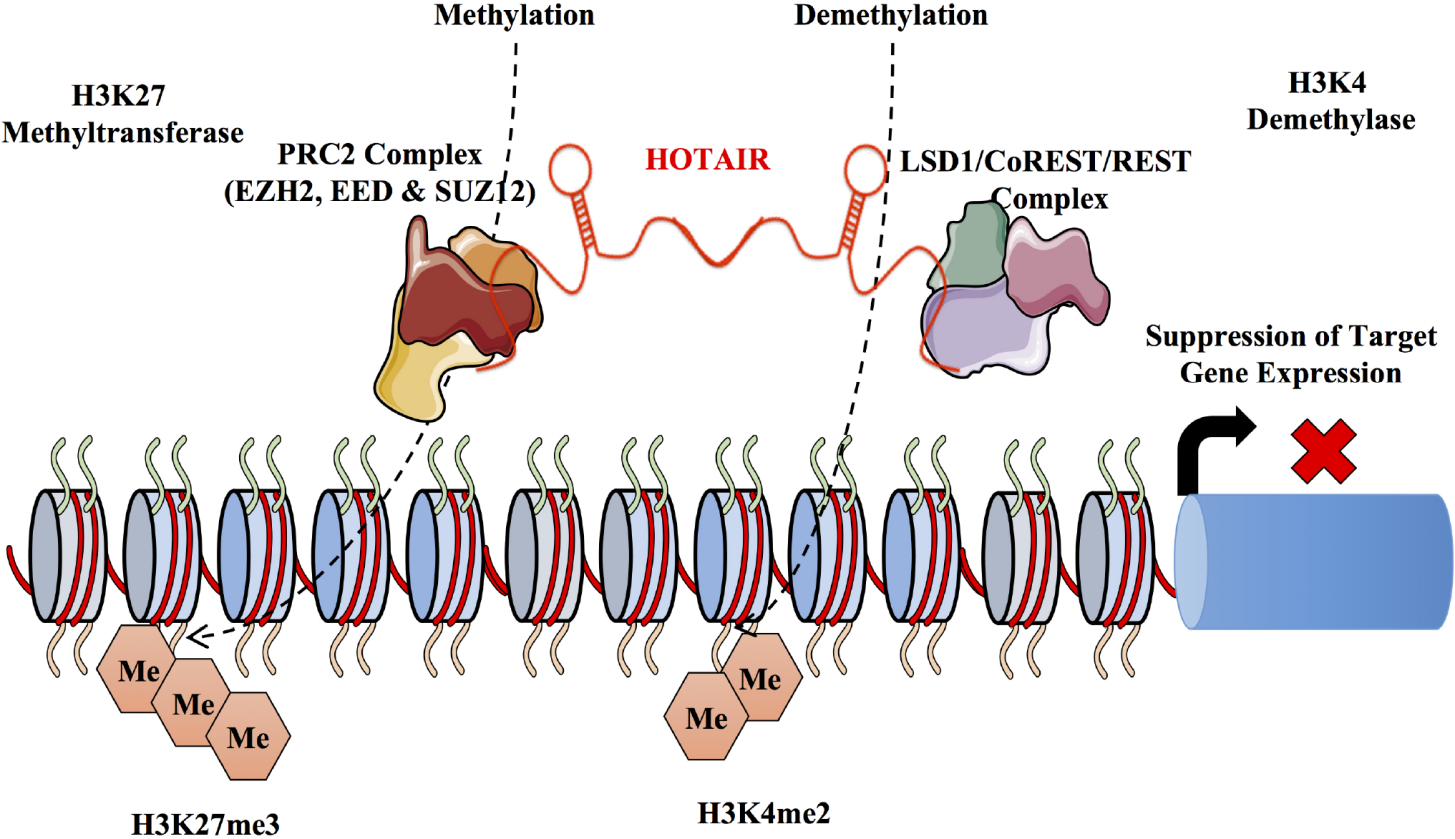
Mechanism of HOTAIR mediated gene silencing The lncRNA HOTAIR is transcribed from the HOXC locus and acts in trans on the HOXD locus. It functions mainly in the recruitment and binding of the PRC2 and the LSD1 complex at the HOXD locus. HOTAIR has a dual function binding the PRC2 complex on the 5` end and the histone lysine demethylase (LSD1) at the 3` end. HOTAIR acts as a molecular scaffold for the protein complexes directing post translational modifications. Interestingly, while PRC2 mediates post translational modifications LSD1 inhibits post translational modifications. HOTAIR is involved in the suppression of metastasis through a series of methylation at the H3K27 and demethylation of the H3K4 histone marks.

HOTAIR is one of the first lincRNAs to be reported and associated with the development of cancer and also the first lincRNA to regulate genes at a distance [186]. As indicated above, HOTAIR though transcribed from the HOXC locus has a more distal mode of action functioning in trans on the HOXD locus accounting for a widespread decrease in expression levels of multiple HOXD target genes [143, 186, 187]. HOTAIR interacts with two key regulators of chromatin dynamics PRC2 and histone demethylase LSD1. Interestingly, HOTAIR functions through a bifunctional mechanism to regulate both proteins. HOTAIR interacts with PRC2 through its 5’ end enhancing repression of PRC2 target loci. In contrast HOTAIR interacts with LSD1 through its 3’ end similarly regulating gene silencing. While PRC2 brings about methylation of the H3K27, LSD1 carries the demethylation of the H3K4 histone marks. Importantly, methylation signatures acquired from tumours exhibiting altered HOTAIR expression have been predicted to function as a biomarker for poor prognosis in various cancers and therapy resistance [188]. HOTAIR has been shown to promote cancer cell proliferation, invasion and metastasis and it is therefore no surprise that HOTAIR has been shown to be overexpressed in several types of cancers [189–192]. Interestingly, HOTAIR is highly expressed in breast cancer compared to normal breast epithelium with recent evidence indicating the increased detection of HOTAIR in peripheral blood mononuclear cells and breast cancer tissues from ER+ and TNBC patients [147]. HOTAIR is also an independent biomarker for predicting the risk of metastasis and mortality in breast cancer with high expression levels of HOTAIR correlating with decreased prognosis. Intriguingly, HOTAIR serves as an independent predictor of metastasis in patients with ER+ breast cancer but not in patients with ER- breast cancer [193]. Experimentally, *in vivo* and *in vitro* overexpression of HOTAIR increases the invasive nature of breast cancer cells. This overexpression of HOTAIR was positively correlated with DNA methylation in primary breast cancer [194]. The process of metastasis involves the coordination of multiple factors including the establishment of epithelial to mesenchymal transition (EMT). In addition, the maintenance of a cancer stem cell population has also been shown to play an important role in cancer metastasis [195, 196]. Interestingly, when HOTAIR is overexpressed, EMT is induced, and the self-renewal capacity of cancer stem cells is maintained [197]. In contrast, upon depletion of HOTAIR both the ability of cells to undergo EMT and overall cancer stem cell populations were diminished. This was further demonstrated in gastric cancer where suppression of HOTAIR reversed the EMT process [198]. The authors go on the show that HOTAIR targets the intercellular adhesion molecule ICAM-1 and members of the matrix metalloproteinase family (MMP1, MMP3 and MMP9) in gastric cancer. MMPs trigger metastasis by remodeling the extra cellular matrix and destroying the basement membrane and when HOTAIR is suppressed MMP1 and MMP3 are also suppressed. HOTAIR also increases the expression of SNAIL, a potent EMT inducer [199]. Furthermore, HOTAIR is involved in the regulation of epithelial cell to cell interactions by increasing the degradation of ATAXIN-1 through ubiquitin mediated degradation, limiting ATAXIN-1 mediated transcription of E-CADHERIN [200–203]. Another way in which HOTAIR can promote metastasis is through its binding to miR 331-3p, resulting the increased expression of HER2. MDA-MB-231 has low levels of HOTAIR expression however, when MDA-MB-231 cells overexpressing HOTAIR were injected in mice tails, an 8-10 fold increase of nodules in the lung was observed suggesting HOTAIR as a positive regulator of metastasis [30, 143]. Similarly, another non-metastatic cell line with low HOTAIR levels SK-BR-3 showed similar effects of metastasis to the lungs but disappeared after a week probably because of its lack of genetic elements allowing tumor persistence in the lungs [143]. Though these initial experiments are indicative of the pro-metastatic properties of HOTAIR the molecular mechanism of how HOTAIR might be involved in EMT and cancer stem cell self renewal might be more complex than thought and further research is undoubtedly required. External factors and exposure to chemical agents can also trigger the differential regulation of lncRNAs. HOTAIR is transcriptionally upregulated by estradiol and is dysregulated by bisphenol-A (BPA) and diethylstilbestrol (DES) [204]. Within the HOTAIR promoter estrogen response elements (ERE) are triggered by BPA and DES through a series of estrogen receptor regulators such as MLL histone methylases (MLL1 and MLL3) which modify the chromatin structure leading to gene activation [204]. This study suggests that the epigenetic status can be altered by exposure to chemical agents leading to a cascade of events such as endocrine disruption. Figure 4 outlines the proposed mechanism for HOTAIR mediated gene silencing in breast cancer.

Along with its role in mammary gland development NEAT1 has also been observed to behave as an oncogene, by promoting proliferation and metastasis in breast cancer patients [205]. LncRNA NEAT1 is highly expressed in breast cancer tissues and expression correlates with tumour size and metastatic potential [205]. RNA immuno-precipitation coupled with high throughput sequencing showed that NEAT1 was associated with FOXN3 and SIN3A repressor complex in ER+ cells. This NEAT1-FOXN3-SIN3A nucleoprotein complex was demonstrated to enhance EMT, invasion and metastasis potentially through the downregulation of the EMT inhibitor GATA3, a direct target of this repressor complex. Interestingly, elevated levels of FOXN3 and NEAT1 correlated with higher histological grade and poorer overall survival in breast cancer [206].

SRA modulates the function of steroid receptors such as estrogen and progesterone along with other transcription factors both at the RNA level and the protein level. This transcript is also highly expressed in numerous cancers including breast cancer suggesting its potential role as a steroid dependent modulator of tumorigenesis. In the aforementioned SRA mouse model, overexpression of SRA lead to an induction of epithelial hyperplasia accompanied by an increase in apoptosis. However, SRA expression itself, is insufficient to promote malignancy [135]. Furthermore, proliferation, inflammation and apoptosis are all induced when SRA is overexpressed in estrogen, progesterone or testosterone sensitive tissues in both male and female mice elucidating to its role in tissue homeostasis in hormone sensitive cells. Early on SRA was thought only to act as non-coding RNA; however, several isoforms of SRA are capable of encoding the endogenous protein (SRAP). The expression of either these two transcripts is controlled by alternative splicing of intron-1, whose retention alters the SRAP reading frame. Nevertheless, it has been observed that both SRAP and the non-coding SRA can co-exist in breast cancer cell lines. Using antisense oligonucleotides to specifically upregulate SRA RNA intron 1 level resulted in marked changes in plasminogen urokinase activator, ERβ, and NME1 expression altering the tumorigenic potential of SRAP knockdown cells. This indicates that altering the balance between the coding and the non-coding SRA transcripts can significantly enhance breast tumorigenesis and tumour progression [207].

BCAR4 was originally identified as a breast cancer oncogene in a functional screen for genes involved in tamoxifen resistance [208, 209]. Interestingly, the lncRNA BCAR4 conferred tamoxifen resistance in a manner independent of the estrogen receptor I expression (ESR1) [209]. Additionally, ectopic expression of BCAR4 in the breast cancer cell lines MCF7 and ZR-75-1 resulted in increased cellular proliferation in estrogen free media. Similarly, BCAR4 induced a growth advantage in the presence of various antiestrogens. BCAR4 was also observed to cause anchorage independent cell growth and promotes metastasis in primary breast tumours [208]. Subsequent xenograft experiments demonstrated ZR-75-1 expressing BCAR4 proliferated more rapidly than control cells when injected in nude mice. Elevated levels of BCAR4 mRNA in patients treated with tamoxifen were associated with reduced progression free survival and clinical benefit and overall survival [210]. BCAR4 overexpressing cells correlated with increased phosphorylation of ERBB2 and ERBB3 suggesting that this pathway may be involved in driving the BCAR4 mediated resistance towards tamoxifen [210]. Interestingly, *in silico* analysis shows that there is a significantly high level of expression of BCAR4 in the human placenta and the oocyte while absent in other normal tissues [209]. BCAR4 may function as a suitable target for treating antiestrogen resistance in breast cancer by harnessing this tissue specific restricted expression pattern displayed by it. In terms of cell migration BCAR4 promotes metastasis which arises from the interaction of chemokines CCL21 and its receptor CCR7, activating the Rho-interacting serine/threonine kinase 21, citron (CIT) which subsequently phosphorylates the transcription factor GLI-2 (Glioma associated oncogene homolog 2) [211, 212]. BCAR4, thus enhances transcription of GLI-2 dependent target genes in breast cancer cells through a non-canonical hedgehog-GLI pathway [213]. BCAR4 also binds to serine /threonine phosphatase regulatory subunit 10 (PSP1R10/PNUTS) and SNIP1 (Smad nuclear interacting protein 1). This interaction of phopsho-GLI2 and SNIP2 releases SNIP1 mediated inhibition of p300 dependent histone acetylation. This downstream signaling leads to binding of PNUTS to H3K18ac leading to the inhibition of PolII via activation of phosphatase PP1. [214, 215]. PP1 eventually dephosporylates RNA polymerase II Ser5 which activates the transcription of GLI2 target genes [216]. Interestingly, locked Nucleic Acids (LNA) against BCAR4 reduced lung metastasis similar to that of of MDA-MB-231 LM2 cells expressing shRNA against BCAR4 showcasing its therapeutic potential [216].

DSCAM-AS1 (Down Syndrome Cell Adhesion Molecule) is a potential oncogenic ER regulated lncRNA, that is upregulated in both ER positive and ER negative cancer compared to normal tissues [217]. Using a cohort of 947 breast cancer patients Niknafs *et al.* probed 58, 648 lncRNAs to systematically profile lncRNA-based expression patterns. Interestingly, the authors demonstrated that like protein expression lncRNAs can stratify breast tumours by their known molecular subtypes. Using this dataset the authors further investigated oncogenic ER-regulated RNAs by comparing lncRNA expression in breast cancer versus normal as well as ER positive versus ER negative comparison [217, 219]. Using this as a cutoff *DSCAM-AS1* was identified as a lncRNA expressed at very high levels in breast cancer tissues, contains ER promoter binding, and exhibits the strongest estrogen induction in both MCF7 and T47D cells. Interestingly, DSCAM-AS1 is an antisense intronic lncRNA that is highly specific for luminal breast cancer. Knockdown of DSCAM-AS1 portrays features similar to the silencing of ERα, such as reduced cell growth, increased apoptosis and the induction of EMT markers. Knockdown of DSCAM-AS1, did lead to the activation of the cell movement. However, another group showed a decrease in migration and invasiveness, which could arise from the silencing strategy used with one being long term silencing and the other transient [217, 218]. Interestingly, ERα expression was not altered by DSCAM-AS1 silencing, suggesting DSCAM-AS1 functions downstream of ERα. Antisense lncRNAs can regulate sense mRNAs at different levels, including transcriptional interference, RNA editing, imprinting, alternative splicing or translation, however, unlike the usual trend DSCAM-AS1 does not regulate the sense mRNA DSCAM [217, 218]. DSCAM-AS1 might thus be associated in breast cancer through the process of transregulation. The unique attributes that lies in DSCAM-AS1 such as its high expression, tissue of origin specificity, and breast tumour phenotype specificity make it an important biomarker of luminal breast cancer. Further investigation needs to be carried out to understand the mechanistic functions of DSCAM-AS1.

Long Intergenic non-coding RNA for Kinase Activation (LINK-A) is a cytoplasmic lincRNA that is significantly elevated in TNBC. LINK-A has recently been observed to be critical for growth factor induced normoxic Hypoxia-inducible Factor α (HIF1α) signaling pathway activation [219]. LINK-A mediates Heparin-binding Epidermal-like growth factor (HB-EGF) dependent EGFR:GPNMB (Glycoprotein non-metastatic b) heterodimerization resulting in HIF1α phosphorylation at Tyr565 and Ser797 by BRK (Breast tumour kinase) and LRRK2 (Leucine-rich repeat kinase 2), respectively. The phosphorylation of Tyr565 interferes with Pro564 hydroxylation leading to HIF1α stabilization while on the other hand, Ser797 phosphorylation enables its interaction with p300, triggering the activation of HIF1α transcriptional programs under normoxic conditions. The activation of HIF1α by LINK-A alters the glycolytic programming in cells enhancing tumorigeneisis. Additionally, LINK-A recruits BRK to the EGFR: GPNMB heterodimer and facilitates the activation of BRK. The correlation between LINK-A and glycolysis in TNBC suggest that LINK-A may be a promising therapeutic target in TNBC [219].

## LNCRNA BASED THERAPEUTICS

It has been proposed that the potential of targeting lncRNAs as a therapeutic strategy may be greater than that of proteins due to their high specificity and their role in specific developmental stages. However, a number of significant drawbacks exist including their sparse availability and overall detection in cancer. This could account for why lncRNA therapeutics have not seen any breakthrough into the clinic. Furthermore, the experimental animal models used may complicate the development of lncRNA therapeutics because of the lack of conservation between species. It is however, worthwhile to mention that the potential use of lncRNA therapies is enormous as it can take advantage of the many functions of lncRNAs. As highlighted in this review lncRNAs can either act in the nucleus or in the cytoplasm and hence determining their expression pattern will be critical in designing effective strategies This can be achieved by strategies like Fluorescent *In Situ* Hybridization (FISH) techniques or more stringent isolation techniques through cellular nuclear and cytoplasmic fractionation [220–223]. Another important factor to consider is the potential functions of the lncRNAs and its relevance to the disease state through a correlation between the lncRNA expression levels and the disease outcomes or symptoms. Subsequently, studying lncRNA gain or loss of function studies by probing for example Fuzzy Kohonen Clustering Networks (FKCN) requires a detailed understanding of specific lncRNA function. Gene silencing strategies also need to be correctly chosen in order to silence lncRNA owing to their localization in the cytoplasm or the nuclei. In general it has been acknowledged that Anti-Sense Oligos (ASO) targeting approaches function better for nuclear lncRNA while duplex RNA approach appear to downregulate cytoplasmic lncRNA with greater efficiency [224, 225].

One such lncRNA predicted to be a therapeutic target is MALAT1. MALAT1 is a nuclear localized lncRNA and targeted inhibition of MALAT1 expression by ASOs significantly reduces tumor growth and metastasis. However, the progression of these compounds to the clinic has been limited by the identification of effective targeting strategies for delivering these ASOs within the tumor cells. Similar to MALAT1, silencing of Survival Associated Mitochondrial Melanoma-Specific Oncogenic ncRNA (SAMMSON), an oncogene which confers a proliferative advantage in melanoma, with ASO gapmers reduced clonogenicity regardless of BRAF, NRAS, or p53 status elucidating to the addiction of melanoma cells to SAMMSON expression. Critically, depletion of SAMMSON had no effect on melanocyte integrity [226]. Another lncRNA, NORAD, is upregulated in response to DNA damage and binds PUMILIO proteins PUM1 and PUM2. Ablation of NORAD using a duplex RNA approach lead to the hyperactivity of PUMILIO genes and to chromosomal instability augmenting the sensibility of NORAD depleted cells to anti-cancer agents. Lastly, Angelman's Syndrome, a severe neurodevelopment disorder is caused by a defect in expression of the maternal gene UBE3A, which encodes an E3 ubiquitin protein ligase. An antisense transcript expressed within the paternal UBE3A locus represses the expression of this allele. ASO targeting of the antisense transcript reversed the cognitive defects in a mouse model [227]. Although, these examples might not necessarily relate to breast cancer pathogenesis or treatment it does highlight the potential of targeting breast cancer specific lncRNAs with either ASOs or duplex shRNA.

## DIAGNOSTIC AND THERAPEUTIC POTENTIAL OF LNCRNA

LncRNAs as mentioned earlier are not only expressed in a tissue and development specific manner, but are also expressed in a cancer type specific manner and hold the potential to be used as prognostic markers. LncRNAs hold the upper hand when it comes to its application as a diagnostic because unlike mRNA, which only provides for an indirect measurement of the functional protein, lncRNAs are a functional end product in itself. Moreover, these functional mature lncRNAs can either enhance post transcriptional modifications or subsequently bind to other proteins. It may not be unrealistic to predict that in the not too distant future lncRNAs may be used as a diagnostic to aid in the pathological diagnosis and classification of certain if not all cancers. With the advent of technology and current diagnostic scenarios, simple quantitative qPCRs leading to more robust and thorough transcriptome profiles generated through RNA sequencing could be employed for the detection of lncRNAs in liquid biopsies. These non-invasive techniques could thus provide for a detailed scenario indicating any abnormal lncRNA levels in the patient. A partial overview of known lncRNAs which have been predicted to act as potential biomarkers is discussed has been discussed in Table 4.

**Table 4:** LncRNA based therapeutic approaches in cancer

Therapeutic agent	Therapeutic approach	Examples of lncRNA
siRNA	Double stranded RNAs. Association with the RISC complex leading to argonaute mediated degradation as a result of perfect sequence similarity.	PANDA
ASO	Single Stranded oligonucleotide sequence with complimentary to the target lncRNA. Effective binding to Secondary structure of lncRNA. RNAse mediated degradation or blocking of translational apparatus.	MALAT1
Ribozymes and deoxyribozymes	Site specific cleaving of RNA by catalytically active RNA or DNA molecules. Targets specific lncRNAs by short sequences about 20 nucleotides long flanking a central loop target.	??
Nucleic Acid Aptamer based	Single-stranded RNA or DNA oligonucleotides capable of efficiently targeting small molecules, peptides, proteins, lncRNA and live cells mediated through 3D structures.	Aptamers CD5, D57, BE9, BC4`, CG3`, DB11 against lncRNA PCA3 (Prostrate Cancer Antigen 3), A30 against HER3
Small Molecule drugs	Compounds that block the activity of target lncRNAs by structure-specific docking to regulate activity.	HOTAIR (Coraline chloride hydrate,Biotin, Ellagic Acid, Camptothecin (S, +)
Locked Nucleic Acids	Modified RNA nucleotide wherein the ribose moiety has an extra bridge linking 2`oxygen and 4` carbon.Works by hybridization, simple base pairing to DNA and RNA.	BCAR4,Miravirsen targeting miR-122
AntagoNATs	Natural antisense transcripts (NATs) can repress mRNA gene expression at the transcriptional level. Targeting NATs with single-stranded oligonucleotides. Blocks interactions of NATs with effector proteins or by RNAse –H degradation of the antisense transcript.	MALAT1

PCA3 has been shown to be a specific biomarker for prostate cancer and act as a negative oncogenic regulator of the tumor suppressor gene PRUNE2 [34, 228]. This has led to the commercialization of a PCA3 assay to permit rapid detection of PCA3 in prostate cancer (Gen-Probe Incorporated, Progensa PCA3 Assay). Similarly, lncRNAs in breast cancer have also emerged as front runners with potential clinical relevance. With respect to breast cancer, HOTAIR and its elevated levels in breast cancer acts as a marker for metastasis [143]. CCAT2 was also shown to be overexpressed and portrays itself as a valuable predictive marker for metastasis free survival and overall survival in patients with local metastasis of lymph node undergoing adjuvant chemotherapy [229]. Loss of GAS5 lncRNA expression shows a strong correlation to resistance of classical chemotherapeutic agents by diminishing anti-proliferative cellular responses including decreased apoptosis when cells are exposed to these agents [230]. A recent report highlighted lncRNA ASBEL as a potential novel therapeutic for TNBC. ASBEL is located antisense of B Cell translocation gene 3 (BTG3), which encodes for an anti-proliferation protein and has been reported to be downregulated in TNBC. The authors demonstrate that knockdown of ASBEL by antisense oligonucleotides re-establishes BTG3 expression resulting in decreased tumor proliferation [231]. Correlating chromatin landscape with lncRNA gene expression data, Su and colleagues demonstrated HOTAIR and HOTAIRM1, are significantly overexpressed in HER2 and TNBC subtypes, respectively [232]. Additionally, a four lncRNA signature of AK024118, BC040204, U79277 and AK000974 could be a potential biomarker in breast cancer patients [233]. Interestingly, investigations from another group showed a dual signature of lncRNA-BC2 and lncRNA-BC5 that were consistently up-regulated while a dual signature of lncRNA BC4 and lncRNA-BC8 that were consistently downregulated in in breast cancer samples [35]. The authors also show a signature or pattern of these lincRNA with respect to their molecular grade [35]. Highly specific and sensitive detection of circulating lncRNAs such as RP11-445H22.4 were found to be elevated in the serum of breast cancer patients [234]. A three lncRNA signature of LINC00324, PTPRG-AS1 and SNHG17, was observed in ER+ and ER- subtypes that were related to their tumour grade, with the first lncRNA significantly up-regulated and the remaining two significantly down-regulated [235]. Furthermore, a number of lincRNAs, lncRNAs, circular RNAs and have been reported earlier to be differentially expressed in breast cancer (refer to Supplementary Table 1) and could have a potential oncogenic or a tumor suppressor effect.

## LNCRNA IN THERAPEUTIC RESISTANCE

Despite advances in early detection and understanding the molecular basis of breast cancer biology, most patients with early stage breast cancer have recurrent disease with progression into metastasis. The heterogeneous nature of the cancer, interspersed with both populations of normal cells and cancer cells makes it a difficult task to specifically target cancer cells. Advancements in the treatment of breast cancer involves the use of cytotoxic drugs, hormonal therapy, targeted therapy and more recently immunotherapeutic agents and are aimed towards the selective administration of less toxic and effective drug treatment, based on the clinical and molecular characteristics of the tumour. In the majority of cases clinical responses are observed however, eventually resistance to these agents occurs. The identification to underlying mechanisms of resistance to these chemotherapeutic agents remains an intense focus of study. Recent efforts have been substantially aided by next-generation sequencing technologies coupled with bioinformatics [236–238]. As part of this, lncRNAs have emerged as important entities in overall drug responses due to their ability to alter the expression pattern of certain genes that might be involved in cell cycle control, apoptosis, or DNA damage and repair pathways [239–242]. We now know that lncRNA expression is widely altered in cancer and participates in various aspects of tumorigenesis through inactivation of tumour suppressors or activation of oncogenes. With respect to breast cancer, increasing reports have cited the importance of lncRNAs in breast cancer drug resistance. Breast cancer progression is mostly dependent on cues provided by estrogen levels with anti-hormone therapy (Tamoxifen) being the major therapy for ER+ breast cancers. Interestingly, a number of lncRNAs have displayed the ability to alter tamoxifen sensitivity. Table 5 shows some of the lncRNAs that are associated with cancer drug resistance and sensitivity.

**Table 5:** LncRNAs associated with breast cancer drug resistance

lncRNA	Drug	Breast cancer /Type	Drug action	Reference
HOTAIR	Tamoxifen	Hormone receptor positive, early and metastatic breast cancers	Attaches on the hormone receptors in cancer cells and blocks estrogen from attaching to the receptor.	[277]
lncRNA-ATB	Trastuzumab	Monoclonal Antibody used for breast cancer that is HER2 positive	Works by binding to HER2 receptor and slows down cell duplication.	[317]
BCAR4	Oestrogen/ Lapatinib	Used in treatment-naïve, ER+/EGFR+/HER2+ breast cancer patients and patients with HER2-positive advanced breast cancer that has progressed after previous treatment to Trastuzumab, anthracycline and taxane derived drugs.	Dual tyrosine kinase inhibitor, that interrupts HER2/neu and EGFR pathways.	[318]
BCAR4	Oestrogen, Tamoxifen	Hormone receptor positive, early and metastatic breast cancers.	Attaches on the hormone receptors in cancer cells and blocks estrogen from attaching to the receptor.	[209, 210]
HIF1A-AS2	Paclitaxel	Used to treat regional or locally advanced breast cancers or secondary breast cancer.	Functions by inhibiting mitotic spindle formation by binding to beta tubulin subunits of microtubules.	[319]
AK124454	Paclitaxel	Used to treat regional or locally advanced breast cancers or secondary breast cancer.	Functions by inhibiting mitotic spindle formation by binding to beta tubulin subunits of microtubules.	[319]
GAS5	Trastuzumab	Monoclonal Antibody used for breast cancer that is HER2 positive.	Works by binding to HER2 receptor and slows down cell duplication.	[270]
UCA1	Tamoxifen	Hormone receptor positive, early and metastatic breast cancers.	Attaches on the hormone receptors in cancer cells and blocks estrogen from attaching to the receptor.	[320, 321]
lncRNA RoR	Tamoxifen	Hormone receptor positive, early and metastatic breast cancers.	Attaches on the hormone receptors in cancer cells and blocks estrogen from attaching to the receptor.	[322, 323]
H19	Paclitaxel	Used to treat regional or locally advanced breast cancers or secondary breast cancer.	Functions by inhibiting mitotic spindle formation by binding to beta tubulin subunits of microtubules.	[324]
CCAT2	Tamoxifen	Hormone receptor positive, early and metastatic breast cancers.	Attaches on the hormone receptors in cancer cells and blocks estrogen from attaching to the receptor.	[325]
HOTAIR	Imatinib and Lapatinib	Imatinib- is used for CML(Chronic Myelogenous Leukemia) and ALL(Acute lymphocytic Leukemia) and other gastrointestinal stromal tumours(GIST). Lapatinib- Used in treatment-naïve, ER+/EGFR+/HER2+ breast cancer patients and patients with HER2-positive advanced breast cancer that has progressed after previous treatment to Trastuzumab, anthracycline and taxane derived drugs. Combinatorial Drug Therapy for Triple Negative Breast Cancer.	Imatinib-Specific inhibitor of tyrosine kinase by occupying the tyrosine kinase active site and decreasing the activity(abl(Abelson proto-oncogene, c-kit and PDGFR(platelet derived growth factor receptor)). Dual tyrosine kinase inhibitor, that interrupts HER2/neu and EGFR pathways.	[326]
LINP1	Radiation and Chemotherapy	LINP1 is overexpressed in triple negative breast cancer. LINP1 enhances repair of the DNA double strand breaks by serving as scaffold liking to Ku-80 and DNA-PKcs LINP1 regulated by p53 and EGFR receptor signalling.	Involved in NHEJ pathway Blocking of LINP1 leads to increased sensitivity of tumour cells to radiotherapy in breast cancer.	[292]

Interestingly, evidence of XIST and another protein 53BP1, have been reported as potential predictive markers of resistance to high dose alkylating chemotherapy and can be used to identify BRCA1-like breast cancer patients. In such a treatment regimen, patients with a high XIST level and low 53BP1 levels were predictors for a poor clinical outcome compared to patients with low XIST levels and high 53BP1 protein level [243, 244]. XIST can also be used as a biomarker predicting breast cancer response to HDAC inhibitors such as abexinostat [245]. The levels of XIST in 16 breast cancer cell lines, led to their grouping into two major classes, one is a low dose sensitive group, in which the cancer stem cells were differentiated by abexinostat and a high dose sensitive group whose cancer stem cell populations were stable [245]. Curiously, in recent experiments carried out in mice low lncRNA XIST expression correlated with cisplatin hypersensitivity, but the mechanism through which this occurs remains elusive [246]. Moreover, XIST can also behave as a tumor suppressor through inhibition of AKT activation in breast cancer [247]. This is achieved mainly by the regulation of HDAC3 via the SPEN/SHARP and SMRT protein complexes. Knockdown of XIST or SPEN, enhanced HDAC3 recruitment to the promoter of PH domain and leucine-rich repeat phosphatase 1(PHLPP1), repressing PHLPP1 expression. PHLPP1 dephosphorylates AKT, converting the active phosphorylated form of AKT to a non-active form decreasing cell viability. Furthermore, another lncRNA, Jpx which is also involved in the tight regulation of XIST and positively regulates it, has decreased levels in breast cancer samples. Subsequently, Jpx knockdown lead to activation of AKT and increased cell viability [247]. These apparent bifunctional roles of XIST in cancer underscores the importance of understanding the molecular mechanisms underlying XIST function in determining when and where targeting XIST would be clinically beneficial. Undoubtedly, further studies will add to this list and it would be unsurprising if the number of lncRNAs does not exceed protein coding transcripts as functional biomarkers for cancer diagnosis and therapeutic sensitivity.

## FUTURE DIRECTIONS, PERSPECTIVES AND FINAL THOUGHTS

Considering the fact that breast cancer is a highly heterogeneous disease centered upon a large number of variables numerous factors are required to be taken into consideration when prediciting therapeutic options. The recent advances in sequencing technology has played a large part in prolonging the survival rates of breast cancer patients but their overall prognosis to date remains limited. Quicker and rapid means of prognosis and diagnosis of breast cancer patients would allow for accurate and efficient therapeutic regimes. The evidence cited in this review point towards an important role of lncRNAs in breast cancer biology and mammary development. LncRNA, being tissue specific and developmental stage specific can be harnessed to serve as potential biomarkers or aid in the prognosis of breast cancer, Additionally, with all but a few exceptions the majority of evidence presented to date on lncRNAs involvement breast cancer is largely based on correlative data where a number of inconsistencies have been reported primarily due to analysis pipelines used for normalization, a diverse set of technology platforms used, the reaction methodologies adapted for sample processing or simply the differences in the sample types, cell lines and tissues analyzed. These discrepancies require follow-up analyses but importantly an international standardization of protocols in respect to tissue collection, processing and data interpretation is required. Only with this standardization can we gain a comprehensive molecular understanding of the roles of lncRNA in respect to breast cancer.

**Figure 5:**
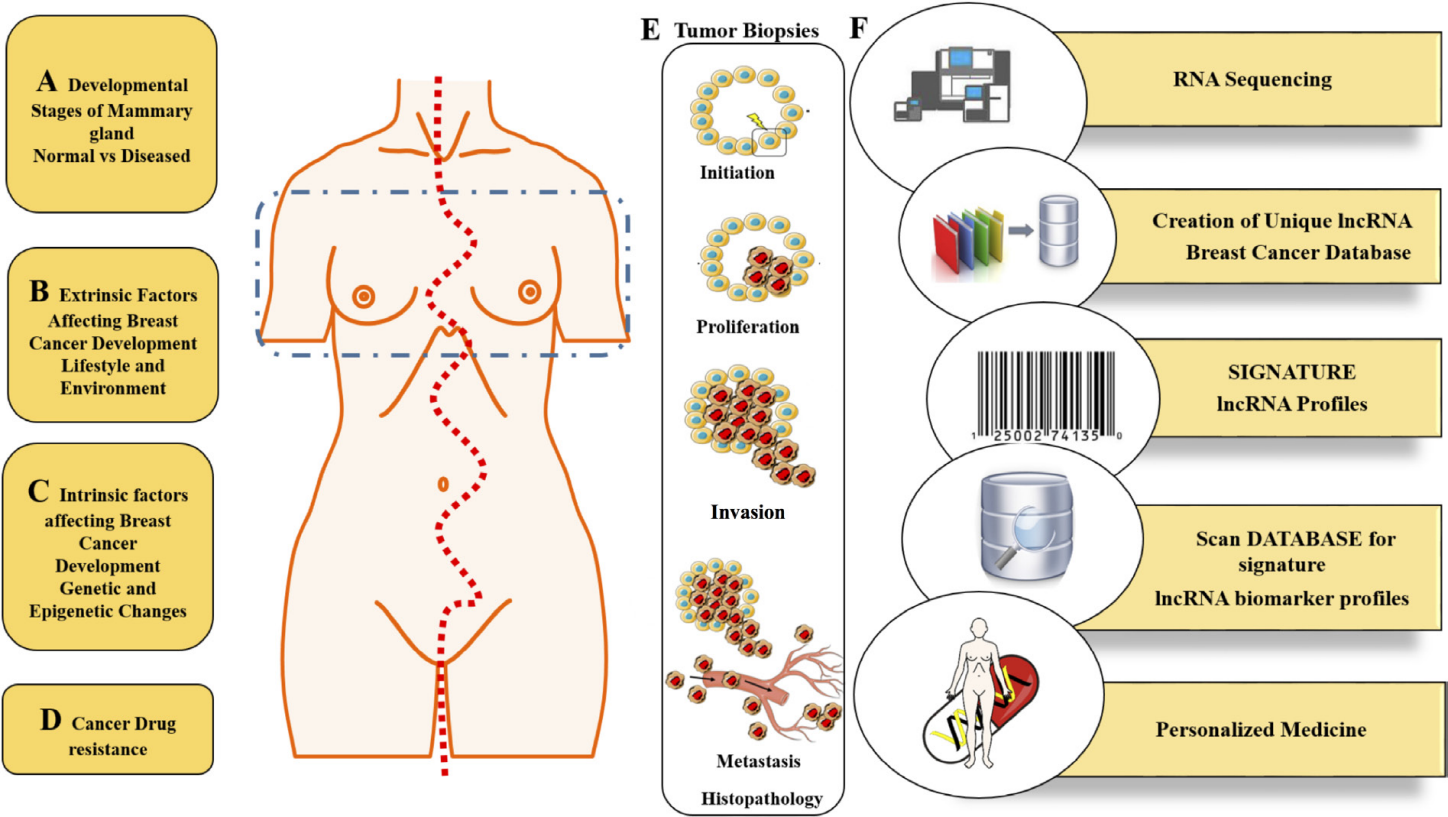
Translational implication of lncRNAs in Breast cancer: What the future holds! Schematic of the diagnostic and therapeutic potential of lncRNAs in breast cancer Presently, breast cancer treatment remains limited by a multitude of factors including genetic heterogeneity and intrinsic or acquired resistance. In these settings breast cancer would greatly benefit from early diagnostic methods to treat breast cancer in its pre-invasive state prior to metastasis. Utilizing lncRNA expression patterns and signatures would ideally aid in this regard. Developmental stages of a normal cell versus a cancer cell can be easily tracked by cataloging the transcriptome profiles of lncRNAs (**A**). Similarly, patients having cancer due to extrinsic factors such as environment and lifestyle as well as intrinsic factors such as epigenetic, heredity and genetic predisposition to the disease would have a unique lncRNA profile (**B** and **C**). Additionally, alterations in the expression pattern of lncRNAs following relapse to targeted therapies may be utilized to understand the mechanism of resistance. (**D**). Linking the lncRNA profiles to the different developmental stages as well the grade/stage of the cancer could help us understand better the cancer scenario and the drug regiment to be administered. A multi-pronged approach including pathological analysis of tumor biopsies along with transcriptomic profiling of both protein coding and non-coding RNA may be beneficial (**E**). Finally, with the advent of technologies and high throughput strategies such as RNA sequencing and transcriptome profiling it would be advantageous to create a unique lncRNA Breast Cancer Database. With the help of statistical and bioinformatics analysis a fine line could be drawn between diseased and normal states through a signature lncRNA profile. In the future, efficient invasive and non-invasive techniques to diagnose breast cancer status will undoubtedly require a comprehensive lncRNA profile of patients providing another cog linking individual disease with verified therapeutic options (**F**).

LncRNAs have nudged their way into the being an important and impacting entity triggering one or more hallmarks of cancer. In this sense, lncRNAs have been reported as important regulators of gene expression and have a broad spectrum of functions including the activation of a diverse set of signaling pathways. The list of such dysregulated lncRNAs in associated disease conditions and normal physiological conditions is on the rise. However, lncRNA research may still be considered in its infancy and is far from being fully recognized due to technical limitations such as its low expression and its fragility as long RNA molecules. A combination of computational analysis along with robust methodologies such as RIP, RAP, CHIRP, CHART, iCLIP and PAR-CLIP followed by sequencing and mass-spectrometry will allow for a detailed understanding of the lncRNA function and mechanism of action in cancer. Keeping in mind the aggressive nature of breast cancer it is important to unearth biomarkers with high sensitivity and specificity to detect breast cancer at an earlier stage. Diagnostic biomarkers that can predict the different stages of breast cancer or yield clinical viable genetic signatures are critical in determining effective treatment therapy options (Figure 5A–5C). Furthermore, resistance to chemotherapy and lncRNAs that are differentially regulated as a result of resistance can suggest alternate clinical treatment approaches (Figure 5D). Identifying lncRNAs as non-invasive biomarkers, that can be robustly detected in liquid biopsies could revolutionize the way breast cancer is detected. The grading of breast cancer through the gold standard procedures of tumor biopsies followed by histological grading can be subjected to transcriptome profiling could reveal important lncRNA signature profiles (Figure 5E, 5F). While lncRNAs still pose a difficulty with cellular targeting, strategies of enhanced delivery using nanotechnology, or exosomes coupled with targeted drug delivery could be beneficial. While this review only covers lncRNAs in breast cancer development, we should not neglect the other non-coding species like miRNAs, piRNAs, tRNA derivatives, snoRNAs and T-UCRs. Unearthing the many functions of non-coding RNAs in cancer development delves into the genomic complexity of cancer and further highlights the extensive interplay between various genetic elements in the cells.

## SUPPLEMENTARY TABLE




