# Supplementary material for: Deciphering the roles of lncRNAs in breast development and disease
Source: Oncotarget. 2018 Feb 28;9(28):20179–212. doi: 10.18632/oncotarget.24591 (PMC5929455; doi:10.18632/oncotarget.24591)
Supplement: Supplementary file 1 [file oncotarget-09-20179-s001.pdf]

Deciphering the roles of lncRNAs in breast development and disease

SUPPLEMENTARY MATERIALS

Supplementary Table 1: Long non-coding RNAs in Breast cancer. See Supplementary_Table_1
