# Supplementary material for: Deciphering the roles of lncRNAs in breast development and disease
Source: Oncotarget. 2018 Feb 28;9(28):20179–212. doi: 10.18632/oncotarget.24591 (PMC5929455; doi:10.18632/oncotarget.24591)
Supplement: Supplementary file 2 [file oncotarget-09-20179-s002.docx]

**Supplementary Table 1. Long non-coding RNAs in Breast cancer**

| **lncRNA** | **Functions** | **Status** | **Reference** |
| --- | --- | --- | --- |
| AK023948 | - Upregulated in breast cancer. - Positive regulator AKT. - Mediated through interaction with DHX9 and p85. - Upregulation of both AK023948 and DHX9 may contribute to breast tumor progression. | Upregulated | [249] |
| AK058003 | - Upregulated in the breast cancer tissues and was found to strongly correlate with breast cancer clinical stage. - AK058003 expression was shown to promote breast cancer proliferation, invasion and metastasis by regulating SNCG expression, Potential biomarker for diagnsosis. | Upregulated | [250] |
| BC040587 | - Potential tumor suppressor. - Low expression levels in breast cancer tissue and breast cancer cell lines. - Overall survival was significantly decreased in patients with low BC040587 expression. | Downregulated | [251] |
| BC200/  BCYRN1 | - Highly expressed in breast cancer tissues and cell lines. - Transcriptionally regulated by estrogen. - Critical for cell proliferation and survival. - BC200 regulated BCL-X- alternative splicing through interaction with hnRNP A2/B1**.** | Upregulated | [252,253] |
| CCAT1 | - Upregulated CCAT1 expression levels correlates with aggressive cancer progression and poor prognosis in breast cancer patients. - cMyc promotes CCAT1 transcription by directly binding to its promoter region. | Upregulated | [254–257] |
| CCAT2 | - Upregulated in breast cancer tissues. - Suppressing CCAT2 expression by siRNA decreases cell proliferation and invasion *in vitro* and inhibits tumorigenesis *in vitro.* - CCAT2 activates the Wnt/β-catenin signalling pathway. | Upregulated | [32,229,258] |
| circ Foxo3 | - Ectopic expression of circFoxo3 represses cell cycle progression by binding to the cell cycle proteins cyclin dependant kinase 2 and cyclin dependent kinase inhibitor 1 ( p21) forming a ternary complex arresting the function of CDK2 and blocking the cell cycle progression. - Additionally blocks the G1-S transition upregulating tumour suppressive protein Foxo3 and acts as a sponge towards apoptosis associated miRNAs such as miR-22, miR-136 and miR-138. | Downregulated | [259,260] |
| circ-ABCB10 | - circ-ABCB10 is significantly up-regulated in breast cancer tissue. - Knockdown of circ-ABCB10 suppressed the proliferation and increased apoptosis of breast cancer cells. - circ-ABCB10 has a sponge effect on miR-1271 and rescued the function of circ-ABCB10 in breast cancer cells. | Upregulated | [261] |
| CUPID1 and CUPID2 | - Predominantly expressed in ER+ breast cancer cells - Estrogen regulated lncRNAs. - Silencing of CUPID1 and CUPID2 leads to inhibition of HR mediated DNA repair. - Play an important role in NHEJ and HDR repair pathway choice. | Upregulated | [262] |
| CYTOR/  LINC00152 | - Required for cell proliferation, cell migration and cytoskeletal organisation. - Upregulated in all types of breast cancer. - Guilt by association analysis highlighted the association between lncRNA and key cancer signalling pathways such as EGFR, mTOR and MAPK pathways. | Upregulated | [263] |
| DANCR | - DANCR expression was increased in Triple negative breast cancer tissues when compared with adjacent normal tissues.using qPCR. - Patients with higher DANCR expression correlated with worse TNM stages as well as shorter overall survival. - Knockdown of DANCR lead to decrease in cell proliferation and cellular invasion in cell lines and reduces tumour growth in *in vivo.* - TNBC cancer stem cell markers ABCG2 transporter, CD44 and ALDH1 were downregulated upon knockdown of DANCR. - Knockdown of DANCR was associated with increased binding of EZH2 on their promoters leading to a reduction in the expression of genes that may be targets of DANCR in TNBC. | Upregulated | [264] |
| DSCAM-AS1 | - DSCAM-AS1 is highly specific for luminal breast cancer. - siRNA of DSCAM-AS1, reduces cell growth, increases apoptosis and induces EMT markers. - Mimics Estrogen receptor-α silencing but does not influence estrogen receptor α expression. - Highly expressed in breast cancer tissues and binds to promoter of estrogen receptor. - Exhibited strong estrogen induction in MCF-7 and T47D cells. - Implicated in Tamoxifen resistance. | Upregulated | [217,218] |
| EPB41L4A-AS2 | - Tumor suppressor by mediating cell proliferation, downregulation promotes tumorigenesis. - EPB41L4A-AS2 associated with tumorigeneis and chemoresistance through regulation of estrogen synthesis. |  | [265] |
| FGF14-AS2 | - Tumor suppressor gene found antisense to FGF14 - Significantly downregulated in breast cancer tissue compared to adjacent normal tissue. - Negatively co-related to tumor size, lymph node metastasis and clinical stage. | Downregulated | [266] |
| GAS5 | - GAS5 is involved in the regulation of apoptosis and differentiation in breast cancer cells. - GAS5 is downregulated in breast cancer and strongly correlated with histological grade and advanced TNM stages. - GAS5 behaves as a riborepressor of the glucocorticoid receptor which regulates cell survival and metabolic activities during starvation. - GAS5 levels are decreased in trastuzumab resistant SKBR-3/Tr cells and patient samples who are undergoing Trastuzumab treatment. | Downregulated | [230,267–270] |
| H19 | - Overexpression promotes tumor progression and is correlated with tumor grade and the presence of ER and PR receptors. - Functions as a myc-upregulated gene promoting tumorigenesis in breast cancer cells. - Important role in development metabolism and cancer by acting as a molecular sponge to regulate members of let-7 miRNA family. - Post-transcriptional level of control by functioning as a precursor of miR-675 and involved in both EMT and MET. - Suppresses expression of E-cadherin and upregulates SLUG. - Overexpression of H19/miR675 enhances breast cancer cell aggressiveness, increased proliferation and migration *in vitro* and increases tumor growth and metastasis *in vivo.* - E3 ligases c-Cbl and Cbl-b act as direct targets of miR-675. | Upregulated | [271–276] |
| HOTAIR | - Upregulated in primary and metastatic tumors. - Acts as repressor in trans by recruiting PRC2 complex and leads to trimethylation of H3K27 at HOXD locus. - HOTAIR binds LSD1 (demethylase of H3K4me2) at 3` domain and EZH2 and SUZ12 (PRC2 components) at 5` end. - Silencing of tumor suppressor genes JAM2 and PCDH. - The TSS of HOTAIR promoter harbours multiple functional estrogen response elements and estrogen co-regulators CBP/p300 and MLL1/3 bind to promoter. - Tumor suppressor gene BRCA1 binds to EZH2 (PRC2 component) and acts as competitive inhibitor of HOTAIR. In cancer states PRC2 binds HOTAIR in reduced BRCA1 conditions to reprogram epithelial cells to cancer cells. | Upregulated | [30,143,186,277–281] |
| HOTAIRM1 | - lncRNA expression correlated with gene expression and chromatin landscape of human mammary epithelial cells and the breast cancer cell line MCF7. - HOTAIRM1 was significantly overexpressed in the basal-like subgroup. - HOTAIRM1 modulates gene expression in both *cis* and *trans.* - Knockdown of HOTAIRM1 results in loss of gene expression of 3`HOXA cluster genes (cis). - Also modulates the β_2_-integrin signalling through CD11b and CD18 and in integrin switch mechanism involving CD11b and CD49d (trans). | Upregulated | [282,283] |
| HULC | - Oncogenic lncRNA upregulated in in TNBC tissues and cell lines. - HULC is correlated with malignant status and poor prognosis of TNBC patients. - Knockdown of HULC supressed TNBC cells migration and invasion. - Also lead to downregulation of MMP-2 and MMP-9 expressions. | Upregulated | [284] |
| LINC00052 | - Positive correlation with HER3/ErbB3 levels in breast cancer cells. - Gene silencing of LINC00052, reduced HER3 level and reduced cancer cell growth *in vitro* and *in vivo* in breast cancer cells and its overexpression produced the exact opposite. - It promotes cancer growth through the HER3 signalling suggestive from experiments carries out with HER3 targeting monoclonal antibodies. - Could serve as a potential biomarker for HER3 targeted antibody cancer therapies. | Upregulated | [285] |
| LINC00324 | - Differentially expressed long intergenic RNA between ER+ & ER- subtypes . - High expression associated with the long survival times of breast cancer patients. | Upregulated | [235] |
| lincRNA RoR | - Highly expressed in breast cancer tissues. - Promotes occurrence and metastasis of breast cancer through EMT. - LincRNA-RoR serves as a competitive endogenous RNA for miR-145, which is critical for TNBC metastasis by targeting ARF6, a known regulator of breast tumor invasion. - Supresses gemcitabine-induced autophagy and apoptosis in MD-MB-231 by silencingmiR-34a expression. | Upregulated | [91,286–288] |
| LINK-A | - Cytoplasmic Intergenic lncRNA. - Elevated levels of LINK-A expression in TNBC. - Critical for growth factor induced normoxic Hypoxia inducible factor α (HIF1 α). - Also required for Heparin binding-epidermal growth factor. - Promotes breast cancer glycolysis reprogramming and tumorigenesis. | Upregulated | [219] |
| lncRNA Hh | - lncRNA Hh is associated with the Sonic hedgehog-GL1 pathway and is transcriptionally regulated by Twist. - It targets GAS1 stimulating hedgehog signalling. - Activated lncRNA Hh leads to the increase in GL1 expression and increases the expression of SOX2 and OCT4 which play an important role in the maintenance of cancer stem cell. - This in turn increases the mammosphere formation efficiency and enhances the self-renewal capacity *in vitro* and the oncogenicity *in vitro* in Twist positive breast cancer cells. | Upregulated | [289] |
| lncRNA HOXA11-AS | - Upregulated in breast cancer models both *in vivo* and *in vitro.* - Knockdown of the lncRNA in MDA-MB231 and MDA-MB-436 inhibited the formation of cell colonies and arrested the cell cycle at the G0/G1 phase. - Suggestive oncogenic role. | Upregulated | [290] |
| lncRNA JADE | - Induced after DNA damage in an ATM-dependent manner. - LncRNA-JADE transcriptionally activates Jade1, a key component in the HBO1 histone acetylation complex. lncRNA-JADE induces histone H4 acetylation in DDR. - Upregulated levels of lncRNA-JADE were observed in human breast tumors. - Knockdown of lncRNA-JADE significantly inhibited breast tumor growth in vivo. | Upregulated | [291] |
| lncRNA LINP1 | - Upregulated in Triple negative Breast Cancer. - Involved in NHEJ pathway and enhances the repair of DNA double strand breaks by serving as a scaffold linking Ku80 and DNA-PKcs. - lncRNA LINP1 is regulated by p53 and epidermal growth factor receptor (EGFR) signalling. - Increases sensitivity to tumour cell response to radiotherapy in breast cancer. | Upregulated | [292] |
| lncRNA LOC | - Elevated in breast cancer tissues and triple negative Breast cancer cell line MDA-MB-231. - Promoted tumorigenesis in breast cancer. - Knockdown decreased cell proliferation, induced apoptosis and impeded tumorigenesis *in vivo.* - Furthermore, LOC554202 is the host gene of miR-31 and eventually dysregulated the hypermethylation at the promoter in triple negative breast cancer. | Upregulated | [293,294] |
| lncRNA-ARA | - ARA expression is significantly associated with adriamycin sensitivity in breast cancer. - Up-regulated in parental sensitive MCF-7 cell lines after receiving adriamycin treatment. - ARA knockdown reduced the proliferation, induced cell death, G2/M arrest and migration defects by modulating multiple pathways. | Upregulated | [295] |
| LSINCT5 | - Overexpressed in breast cancer cells and tissues. - Knocking down the expression of LSINCT5 decreases cellular proliferation in cancer derived cell lines. | Upregulated | [296] |
| MALAT1 | - Downregulated in breast cancer cell lines as well as breast cancer tissue. - MALAT-1 regulates metastasis by inducing EMT via activation of the PI3K pathway. - MALAT-1 is linked to overall survival. - Behaves as an oncogene in TNBC particularly through its interaction with miR-1 and cdc42. - Treatment of TNBC cell lines with high concentration of estradiol decreased MALAT-1 RNA levels by posttranslational modification in an ERα independant manner. | Downregulated | [31,297–303] |
| MaTAR | - 19 Mammary Tumour Associated RNAs were identified that were significantly upregulated in breast cancer correlating with their hormone receptor status and cancer subtype. - Knockdown of MaTARs lead to decrease in cell viability and invasion. - The knockdown additionally inhibits collective cell migration in organoids. | Upregulated | [304] |
| MEG3 | - Maternally expressed gene 3 (MEG3) is an imprinted gene and a potential tumour suppressor. - Overexpression of MEG3 inhibits tumour cell proliferation - It also leads to accumulation of p53. - MEG3 modulates the activity of TGFβgenes. | Downregulated | [305,306] |
| MVIH | - Elevated expression levels of MVIH influences cell proliferation, apoptosis and cell cycle. - Correlated with high Ki67staining, poor overall and disease free survival. | Upregulated | [307] |
| NBAT-1 | - Downregulated in patients who received hormone replacement therapy. - Negative correlation with estrogen and progesterone receptors. - Acts as tumor suppressor by binding to PRC2 complex component EZH2. - Low NBAT-1 expression levels in breast cancer are associated with poor survival and development of lymph node metastases. - NBAT-1 upregulates DKK1 therein supressing migration and invasion of breast cancer cells. | Downregulated | [80] |
| NEAT1 | - NEAT1 is regulated principally by HIF-2 rather than by HIF-1. - Induction of NEAT1 in hypoxia also leads to accelerated cellular proliferation, improved clonogenic survival and reduced apoptosis. - High tumor NEAT1 expression correlates with poor survival in breast cancer. - Downregulation of NEAT1 in breast cancer cells inhibited cell growth and induced apoptosis. - In addition, the RNA-binding protein FUS/TLS physically interacted with NEAT1. | Upregulated | [19,308] |
| NKILA | - NKILA is upregulated by NF-kB and binds to NF-kB/IkB to form a stable complex. - NKILA blocks the IkB phosphorylation and behaves as a NF-kB modulator.-Decreased NKILA expression is associated with breast cancer metastasis and poor patient prognosis. | Upregulated | [309] |
| PANDAR | - Upregulated in breast cancer tissues and breast cancer cell lines and behaves as oncogene by regulating G1/S transition during cell cycle by supressing p16^INKA^. - Expression of PANDAR induced by p53 upon DNA damage. - Interacts with NF-YA to supress pro-apoptotic genes and enables cell cycle arrest. | Upregulated | [219] |
| PTPRG-AS1 | - Differentially expressed antisense RNA between ER+ & ER- subtypes - Low expression associated with the long survival times of breast cancer patients | Downregulated | [235] |
| SNHG17 | - Differentially expressed small nucleolar RNA host gene between ER+ & ERsubtypes. - Its expression in breast cancer correlates with tumor grade. - Low expression associated with the long survival times of breast cancer patients. | Downregulated | [235] |
| Spry4-ITI | - SPRY4-IT1 expression corresponded with tumor size and later stage of tumor development. - siRNA of SPRY4-IT1 suppressed cell proliferation and induced apoptosis in breast cancer cells. - ZNF703 identified a target of the lncRNA promotes proliferation and supressed apoptosis *in vivo.* | Upregulated | [310] |
| TALAM1 | - TALAM1 is a Natural Antisense Transcript that positively regulates MALAT1 by promoting the 3` end cleavage and maturation of MALAT1 RNA both at the level of RNA stability and in the level of transcription. - TALAM1 not only interacts with MALAT1 but is also found localised in the site of transcription. - Overexpression of TALAM1 facilitates the cleavage reaction in trans while its depletion led to defects in the 3` end cleavage reaction of MALAT1 and leads to its accumulation. | ?? | [311] |
| uc.63 | - Associated in patients with luminal A breast cancer. - Silencing uc.63 induces apoptosis. - Localised in Exportin-1 Gene (XPO1) and transcribed in same orientation. - Silencing experiments on XPO1 show that uc.63 is independent of host gene and promotes survival of breast cancer cells. | Upregulated | [312,313] |
| UCA1 | - Highly expressed in breast cancer. - Behaves as a microRNA sponge (*miR-193a-3p-sponge*) in NSCLC cells when upregulated. - In breast cancer UCA1 modulates cell growth and apoptosis by interacting with *miR-143*. - UCA1 supresses tumor suppressor p27 through interaction with heterogeneous nuclear ribonuclearproteins 1 (hnRNP1). - Knockdown of UCA1 increases tamoxifen resistance through inhibition of Wnt pathway. | Upregulated | [314–316] |
